# Macroecology of Abiotic Stress Tolerance in Woody Plants of the Northern Hemisphere: Tolerance Biomes and Polytolerance Hotspots

**DOI:** 10.1111/ele.70016

**Published:** 2024-12-02

**Authors:** Nicola Pavanetto, Ülo Niinemets, Marta Rueda, Giacomo Puglielli

**Affiliations:** ^1^ Institute of Agricultural and Environmental Sciences Estonian University of Life Sciences Tartu Estonia; ^2^ Estonian Academy of Sciences Tallinn Estonia; ^3^ Departamento de Biología Vegetal y Ecología, Facultad de Biología Universidad de Sevilla Sevilla Spain

**Keywords:** abiotic stress, adaptive strategies, polytolerance, stress biomes, woody plants

## Abstract

Understanding the main ecological constraints on plants' adaptive strategies to tolerate multiple abiotic stresses is a central topic in plant ecology. We aimed to uncover such constraints by analysing how the interactions between climate, soil features and species functional traits co‐determine the distribution and diversity of stress tolerance strategies to drought, shade, cold and waterlogging in woody plants of the Northern Hemisphere. Functional traits and soil fertility predominantly determined drought and waterlogging/cold tolerance strategies, while climatic factors strongly influenced shade tolerance. We describe the observed patterns by defining ‘stress tolerance biomes’ and ‘polytolerance hotspots’, that is, geographic regions where woody plant assemblages have converged to specific tolerance strategies and where the coexistence of multiple tolerance strategies is frequent. The depiction of these regions provides the first macroecological overview of the main environmental and functional requirements underlying the ecological limits to the diversity of abiotic stress tolerance strategies in woody plants.

## Graphical abstract

We provide the first macroecological overview of the main environmental and functional requirements underlying the diversity of abiotic stress tolerance strategies in woody plants of the Northern Hemisphere by defining ‘stress tolerance biomes’ and ‘polytolerance hotspots’, that is, geographic regions where woody plant assemblages have converged towards specific tolerance strategies and where coexistence of multiple tolerance strategies is frequent.

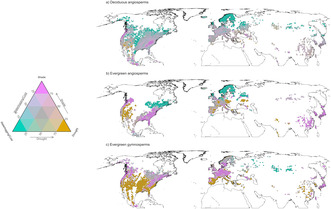

## 1 Introduction

Climate change is altering the severity of abiotic stresses and their interactions, exerting negative impacts on plant performance at all scales (Niinemets 2010; Zhang et al. 2018). This phenomenon is of particular concern in the case of long‐living organisms such as woody plants, which constitute most of the global standing forest biomass and are key components of terrestrial carbon storage (Bonan 2008). Long‐term interactions among different levels of abiotic factors such as temperature, light and water availability in a species habitat are recognised as the main drivers of the adaptive evolution of woody plant tolerance strategies to multiple abiotic stressors (Valladares and Niinemets 2008; Kunstler et al. 2016; Puglielli, Hutchings, and Laanisto 2021; Puglielli, Laanisto et al. 2023). Along broad environmental gradients, the interactions among levels of temperature, light and water availability ultimately define a complex landscape of diversity of stress tolerance strategies (Hawkins et al. 2014; Puglielli, Tordoni et al. 2023). However, the determinants of this diversity and its distribution pattern remain largely unexplored.

**FIGURE 1:**
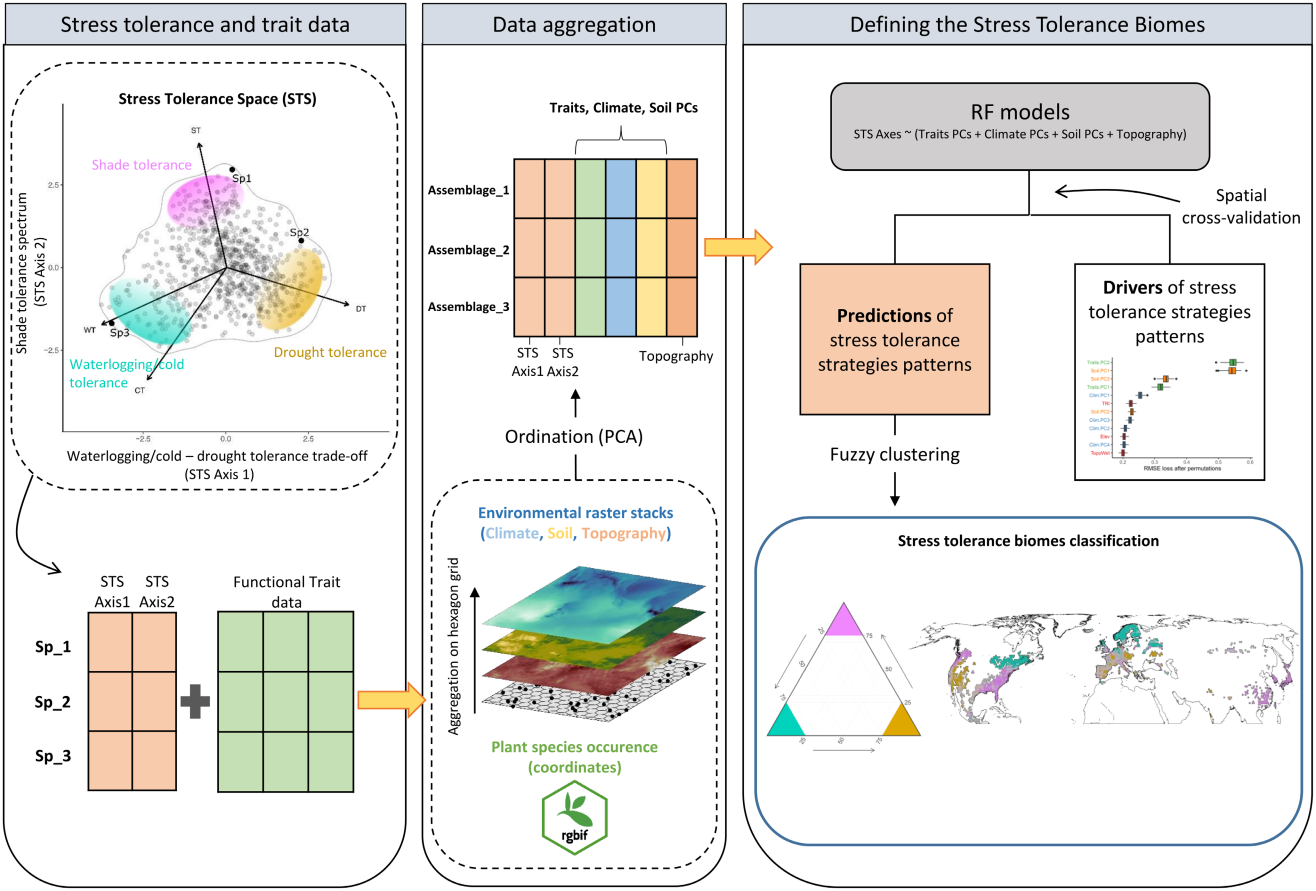
Schematic representation of the workflow. Stress tolerance data were obtained from the stress tolerance space (STS) framework. The STS is defined by combinations of tolerance to drought (DT), shade (ST), waterlogging (WT) and cold (CT) for 799 species in the Northern Hemisphere. The first axis reflects a trade‐off between drought and waterlogging/cold tolerance, while the second axis is a shade tolerance spectrum, from low to high shade tolerance. The STS axes values for each species were associated with the functional traits of each species and then spatially aggregated in a hexagonal grid based on species occurrence. For each hexagon of the grid, the mean values of climate, soil and topographic variables were calculated as environmental predictors. Functional traits, climate and soil variables were subjected to principal component analysis (PCA) to reduce dimensionality and account for correlation among variables. The resulting PCs were used as predictors in random forest models to generate environmentally informed spatial predictions of stress tolerance strategies and to identify characteristics of stress tolerance strategy patterns. By grouping the stress tolerance predictions with a fuzzy clustering algorithm, we identified areas where communities have converged on specialised stress tolerance strategies towards drought, shade and waterlogging/cold.

Across the Northern Hemisphere, the interactions between abiotic stresses, as well as their severity and timing, widely differ among forested ecosystems (Harfouche, Meilan, and Altman 2014). Despite the potential diversity of resulting tolerance strategies, trade‐offs among tolerances to multiple stressors shape the realised multi‐stress tolerance strategies of woody plants (Laanisto and Niinemets 2015; Niinemets and Valladares 2006; Puglielli, Hutchings, and Laanisto 2021; Puglielli, Laanisto et al. 2021; Puglielli, Tordoni et al. 2023). As a result, across woody plants of the Northern Hemisphere, strategies to tolerate four key abiotic stressors (shade, drought, cold and waterlogging) are summarised along two main tolerance dimensions (Puglielli, Hutchings, and Laanisto 2021). One dimension is a trade‐off between drought and tolerance to waterlogging/cold. The second dimension is a shade tolerance spectrum that adds an independent tolerance dimension to the drought‐waterlogging/cold trade‐off. Tolerance combinations along these two dimensions define a triangular space, hereafter the stress tolerance space (STS, Figure 1), where pairs of coordinates reflect realised species‐specific multi‐stress tolerance strategies to shade, drought, waterlogging and cold tolerance for woody plants of the Northern Hemisphere. At the tips of the STS, trade‐offs among tolerances are stronger, and these regions are mostly occupied by either drought, shade or waterlogging/cold‐tolerant species. Along the sides of the triangular space and between vertices, trade‐offs among tolerances are looser, and these regions are occupied by species with tolerance strategies characterised by a relatively high tolerance of two or more stressors (hereafter polytolerance strategies, *sensu* Niinemets and Valladares 2006). In sum, the STS provides us with a general framework to understand the main axes of variation in tolerance strategies of woody plants throughout the Northern Hemisphere. The key question is what determines the relative position of the species within the STS. Answering this question has the potential to clarify the nature of the trade‐offs limiting the diversity of tolerance strategies of Northern Hemisphere woody plants.

The position of the species within the STS must ultimately be explained by species' functional trait syndromes and by the soil and climatic factors that jointly select for such syndromes (Puglielli, Pavanetto, and Laanisto 2022). Trait‐based ecology has provided valuable insights into the joint influence of climate and soil drivers on species morpho‐functional features along environmental gradients at multiple scales (Wright et al. 2004; Reich 2014; Simpson, Richardson, and Laughlin 2016; Bruelheide et al. 2018; Bjorkman et al. 2018; Tordoni et al. 2022). Recently, a global study (Joswig et al. 2022) revealed that climatic and soil factors account for 77% of trait variation within the Global Spectrum of Plant Form and Function (Díaz et al. 2016). Variations in size‐related traits (plant height, seed mass, specific stem density, leaf size) were correlated with a latitudinal gradient of water and energy availability, while leaf economic traits (specific leaf area, nitrogen content per dry mass) were primarily explained by interactions between climate and soil fertility. In particular, the variation in leaf economic traits was primarily driven by organic matter concentration and soil pH (Joswig et al. 2022). Recently, trait syndromes defining the global spectrum of plant form and function were also successfully used to explain key functional constraints on abiotic stress tolerance strategies in woody plants as defined by their position in the STS (Pavanetto et al. 2024). Hence, we can leverage the framework linking the trait syndromes that define the global spectrum of plant form and function with their inherent climate and soil drivers (Joswig et al. 2022) to uncover the current distribution of tolerance strategies in woody plants of the Northern Hemisphere and finally describe the large‐scale ecological constraints underlying tolerance trade‐offs.

Our main objectives were (i) to identify the main descriptors of the diversity of woody plant tolerance strategies to multiple stressors across the Northern Hemisphere and (ii) to map the spatial distribution patterns of abiotic stress tolerance strategies of woody plants across the Northern Hemisphere. Our data set included abiotic stress tolerance strategies as defined by the STS framework, climatic/edaphic conditions in species habitats and functional traits of the global spectrum of plant form and function. This multidimensional approach allowed us to identify geographic areas sharing similar climatic, soil, and functional characteristics where communities have converged on specialised stress tolerance strategies, thereby introducing the concept of abiotic stress tolerance biomes (STBs, Figure 1). Furthermore, by leveraging the possibility of identifying relative polytolerance areas in the STS (see above), we also identified polytolerance hotspots, that is, geographical regions where communities have adapted to maximise either shade‐drought or shade‐waterlogging/cold polytolerance.

## 2 Methods

### 2.1 Stress Tolerance Data

Species‐specific tolerance strategies defined by the combinations of drought (DT), cold (CT), waterlogging (WT) and shade (ST) were retrieved from the Stress Tolerance Space (STS) data set (Puglielli, Hutchings, and Laanisto 2021). The STS was defined using published data on woody plant tolerance to abiotic stress (Niinemets and Valladares 2006; Laanisto and Niinemets 2015) and it summarises species‐specific combinations of tolerance to the four mentioned stressors for 799 species of the Northern Hemisphere spanning three major plant functional types: deciduous angiosperms (*n* = 547), evergreen angiosperms (*n* = 134) and evergreen gymnosperms (*n* = 106). The STS analysis revealed two primary independent tolerance dimensions that explained 72% of the total variance in the stress tolerance data. The first dimension represents a trade‐off between waterlogging/cold and drought tolerance (hereafter, waterlogging/cold‐drought trade‐off), while the second dimension represents a shade tolerance spectrum (Puglielli, Hutchings, and Laanisto 2021). Species‐specific STS coordinates are available in Puglielli, Hutchings, and Laanisto (2021). The species nomenclature follows The Plant List v.1.1 (www.theplantlist.org).

### 2.2 Trait Data

The traits defining the Global Spectrum of Plant Form and Function (GSPFF, Díaz et al. 2016, see Introduction)—maximum plant height (PH, m), specific stem density (SSD, mg mm^−3^), seed mass (SM, mg), individual leaf size (LA, mm^2^), leaf nitrogen content per unit of leaf dry mass (LN, mg g^−1^) and specific leaf area (SLA, mm^2^ mg^−1^) were obtained for 779 species in the STS from Carmona et al. (2021) who compiled trait data from public data sets in the TRY Plant Trait Database (version 5.0, https://www.try‐db.org/TryWeb/Prop2.php, Kattge et al. 2020). Combinations of these traits reflect mechanical and energetic trade‐offs that constrain aboveground vascular plant functional diversity at the global scale, and they are relevant for defining the trait syndromes underlying woody species' abiotic stress tolerance strategies within the STS (Pavanetto et al. 2024). For our analyses, we used a previously imputed version of the trait data set that was already carefully tested in terms of robustness in defining the trait dimensions underlying the abiotic stress tolerance strategies in the STS (Pavanetto et al. 2024).

### 2.3 Species Occurrence Data

We obtained species occurrences from the Global Biodiversity Information Facility (GBIF, GBIF.org, last accessed on 18/10/2022) using the R package ‘rgbif’ (Chamberlain et al. 2022). We used the ‘backbone method’ provided by ‘rgbif’ to update the species names to the latest classification based on The Plant List database to match the nomenclature with the STS data set. Occurrence data for each species were collected considering only their native continental origin (i.e., North America, Europe‐Western Asian and East Asia), as defined by Niinemets and Valladares (2006). Although GBIF has an irreplaceable value, available occurrence data suffer from several inherent limitations (Supporting Information Note S1). To ensure the best quality for the GBIF data included in the analysis, the raw occurrence were rigorously cleaned and validated using multiple approaches detailed in Note S1 (i.e., coordinate validity environmental and geographical filtering). This approach mitigated potential biases such as the uneven distribution of occurrences within the environmental space derived from species occurrences (Varela et al. 2014; Castellanos et al. 2019).

### 2.4 Climatic and Soil Variables

The final occurrence data were used to query 22 climatic variables included in the CHELSA v2.1 database (www.chelsaclimate.org, accessed on 17/01/2023) at a resolution of 30 arcsec. The use of CHELSA variables has been shown to result in greater predictive power in species distribution models compared to the widely used WorldClim variables (Fick and Hijmans 2017), particularly in topographically complex and heterogeneous regions (Maria and Udo 2017). From CHELSA, together with bioclimatic variables, we also obtained variables summarising the length of the growing season and altitude (based on the global Digital Elevation Model, DEM, used by CHELSA). The climatic variables used in this study are reported in Table S1.

To characterise soil conditions, we retrieved eight soil variables from the SoilGrid dataset (https://soilgrids.org/) at a 30 arcsec resolution using the function *soil_world* from the ‘geodata’ R package (Hijmans et al. 2023). SoilGrid provides global predictions of various soil characteristics at depth intervals of 0–5 cm, 5–15 cm, 15–30 cm, 30–60 cm, 60–100 cm and 100–200 cm. For each soil variable, we calculated the mean values across the first four depth intervals (Hengl et al. 2017). Finally, to incorporate information on topography, we downloaded layers for topographic wetness index (TWI) and topographic roughness index (TRI) from the ENVIREM database (Title and Bemmels 2018) at a resolution of 30 arcsec. TWI and TRI quantify terrain‐driven variations in soil moisture and topographic heterogeneity, respectively. The edaphic and topographic variables used in this study are reported in Table S1.

The retrieved climate and soil information was then aggregated in equal‐area hexagons (7500 km^2^ each) using standard procedures (detailed in Note S2). Our final data set included 2791, 1919 and 2245 hexagons for deciduous angiosperms, evergreen angiosperms, and evergreen gymnosperms, respectively, covering 21%, 14.4% and 16.9% of the Northern Hemisphere. The need to differentiate between these plant functional types (PFTs) stems from their different distributions in both geographic space and STS (Figure S2, Table S3).

### 2.5 Statistical Analysis

#### 2.5.1 Descriptors of Abiotic Stress Tolerance Strategies

To identify the features that describe abiotic stress tolerance strategies in our data set, we fitted random forest (RF) models using the waterlogging/cold‐drought tolerance trade‐off and the shade tolerance spectrum (i.e., the STS axes) as response variables. While RF models can handle overfitting and multicollinearity (Cutler et al. 2007; Mansfield et al. 2020), highly correlated predictors can still lead to incorrect conclusions when interpreting variable importance (Strobl et al. 2007). Thus, after standardising and centring the mean environmental and log‐10 transformed trait variables for all the hexagons, we conducted three separate principal component analyses (PCA) for the climate, soil, and trait data (Note S3, Table S4, Figures S3–S5). The number of principal components (PCs) retained was defined using the *paran* function in the ‘paran’ R package (Dinno 2018) that uses the Horn's parallel analysis to identify the optimal number of PCA axes to be retained (Dinno 2018). The retained PCs exhibited weak correlations with each other (Pearson's *r* always < 0.54, Figure S6). To facilitate the interpretation of the results, we applied a varimax rotation to the principal components. Varimax rotation changes the coordinates in the PCA to maximise the sum of the variances of the squared loadings, without changing the overall relationships between the variables (Abdi and Williams 2010) and without distortions (Bueno et al. 2023).

We used the retained PCs of each climatic, soil and trait space as predictors in the RF models (Figure 1). We added elevation, topographic roughness index and topographic wetness index as additional variables. We did not explicitly include latitude or longitude in the models, since their inclusion often leads to overfitting and incorrect estimation of variable importance when dealing with spatial data (Meyer et al. 2019). However, we tested the effect of latitude and longitude on the spatial cross‐validated RF models' residuals (see Note S4) to conclude that our models well captured the spatial structure of the data (Table S5). A total of six RF models were trained, with either the waterlogging/cold‐drought trade‐off or the shade tolerance spectrum (i.e., STS axes) as the response variable. The models were executed individually for each PFT due to their different distributions and trait syndromes. Details on model specifications along with uncertainty maps are reported in Note S4, Figures S7 and S8. The relative importance of the variables was evaluated by estimating the average change in the root mean squared error (RMSE) after permuting the variables (*n* = 500) using the ‘DALEX’ R package (Biecek, Maksymiuk, and Baniecki 2023). This method assumes that when an important variable is permuted, this should worsen the overall model performance due to a loss of explanatory ability (i.e., RMSE loss after permutation, Breiman 2001). Partial dependence plots were generated to visualise the marginal effects of the predictors.

#### 2.5.2 Defining the Stress Tolerance Biomes and Polytolerance Hotspots

The predictions from RF models were used to classify hexagons into groups with similar tolerance characteristics by PFT. We identified groups of hexagons using a fuzzy *K*‐means cluster analysis. This analysis allows classification of data points that belong to more than one cluster by assigning a degree of membership to a given cluster (ranging from 1, indicating perfect assignment, to 0, denoting complete exclusion). Fuzzy *K*‐means clustering was performed using the *FKM* function from the ‘fclust’ package (Giordani, Ferraro, and Serafini 2022), using a fuzzy silhouette approach (Campello and Hruschka 2006) to estimate the optimal number of clusters over 99 repetitions. This process identified four distinct groups (hard clusters) with shared stress tolerance characteristics (Figures S9 and S10). Since we were especially interested in the tip‐level strategies within the STS, that is, shade, drought and waterlogging/cold tolerance strategies (Figure 1), we removed the column relative to the low‐intermediate tolerance cluster from the original membership degree matrix and rescaled the remaining values by their sum to obtain a ternary scheme. Note that when this transformation is applied, given the triangular structure of the STS, the low‐intermediate tolerance strategy is still represented by relatively low membership values to the three remaining clusters. The rescaling allowed us to realistically reflect the triangular structure of the STS. In addition, this approach allowed us to identify the assemblages that display polytolerance, namely the assemblages with split membership degree (0.4–0.6) toward two end‐point strategies (either shade–drought or shade–waterlogging/cold).

We then characterised the regions corresponding to the three main tolerance and the two polytolerance strategies using the associated climate, soil and functional traits and defined these regions as Stress Tolerance Biomes (STB) and polytolerance hotspots, respectively. Climate, soil and functional traits information for each STB and polytolerance hotspot was defined by matching them with the principal components of climate, soil and functional traits already described by using the ‘funspace’ R package (Carmona, Pavanetto, and Puglielli 2024).

However, given the relatively low sample size of polytolerant assemblages compared to that of tip‐level ones, together with the arbitrary choice of the cluster membership threshold to define polytolerance regions, we preferred to express polytolerance hotspots in a probabilistic fashion to account for potentially greater uncertainty in their definition compared to the STBs. This was done using the *kde* function in the ‘SpatialKDE’ R package (Caha 2023), which allows kernel density estimation with a user‐defined spatial bandwidth (Hart and Zandbergen 2014). We used a radius of 711,000 Equal Earth projection units (corresponding to ~10,000 km^2^) for the spatial bandwidth with a triweight kernel function to discriminate different hotspots.

Finally, we characterised each STB and polytolerance hotspot in terms of potential natural vegetation (PNV)—vegetation cover in equilibrium with climate and unaffected by human activities (Hemsing and Bryn 2012)—by calculating the cumulative overlap percentage of the area of each PNV in each biome and hotspot. PNV information was obtained as a global map at 1 km resolution (Hengl et al. 2018) based on an expanded version of the BIOME 6000 dB classification (i.e., megabiomes, following Harrison 2017). Additionally, we applied the described procedure also including an alternative biome classification, that is, the ecoregions by Dinerstein et al. (2017). All analyses were performed using R version 4.2.2 (R Core Team 2022).

## 3 Results

### 3.1 Descriptors of Stress Tolerance Strategies

**FIGURE 2:**
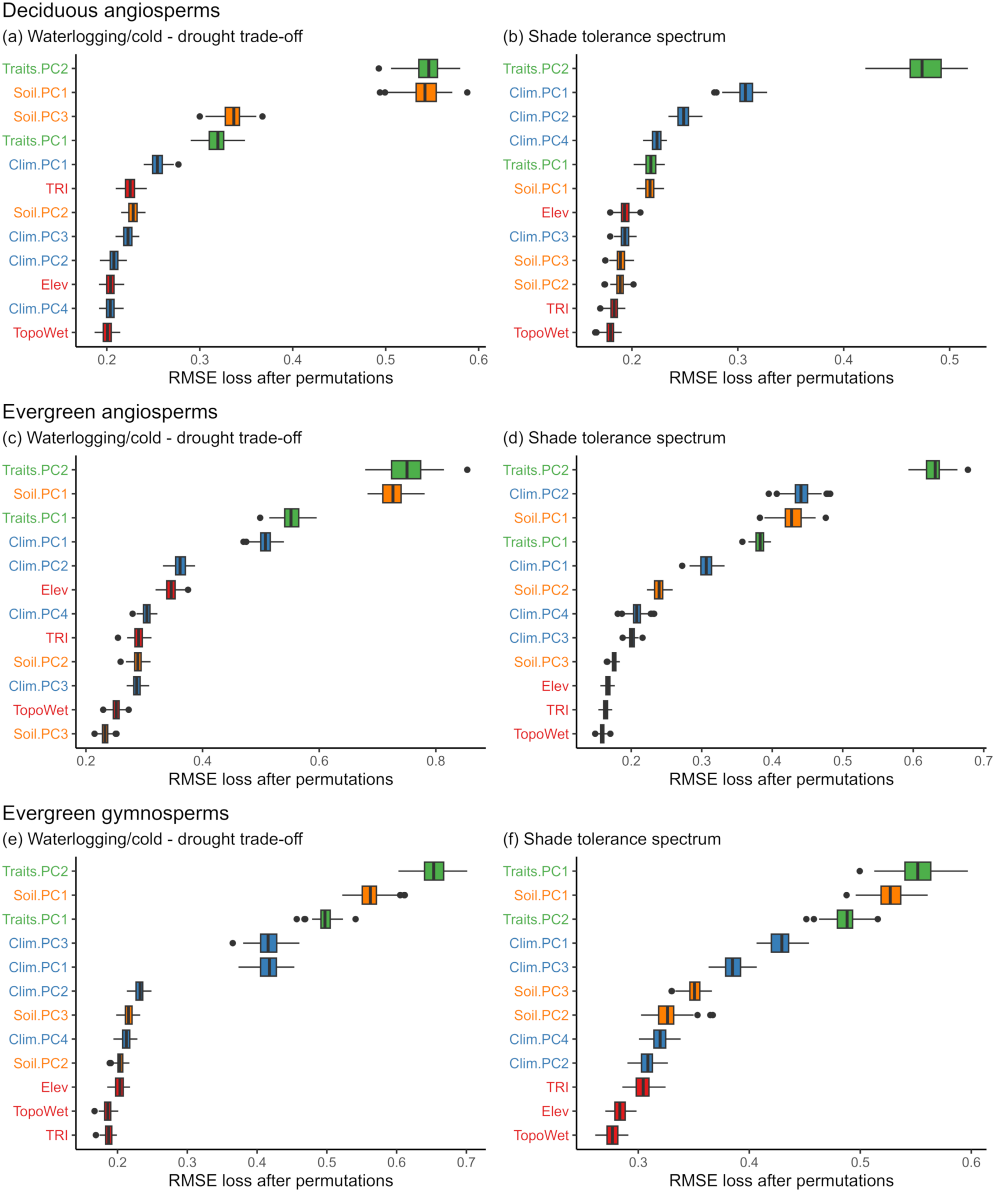
Variable importance of the selected descriptors of the STS axes. The waterlogging/cold‐drought tolerance trade‐off and the shade tolerance spectrum for deciduous (a, b) and evergreen angiosperms (c, d) and evergreen gymnosperms (e, f). Boxplots show variable importance ranked by the root mean squared error (RMSE) loss‐function after permutation (see Section 2.5—Assessing the features of abiotic stress tolerance strategies section). This measure quantifies the worsening of the model (i.e., increase in RMSE) when a variable is permuted. Green labels indicate trait predictors, orange labels indicate soil predictors, and blue labels indicate the climate predictors; red labels indicate the topographic roughness index (TRI); elevation (Elev) and topographic wetness index (TopoWet).

The trait dimensions (Traits.PC1 and Traits.PC2, Note S3) emerged as the most important predictor across most PFTs and for both the drought‐waterlogging/cold trade‐off and the shade tolerance spectrum (Figure 2a–f). The drought‐tolerant assemblages of both angiosperms and gymnosperms exhibited higher values along Traits.PC2, corresponding to higher specific stem density (SSD), seed mass (SM), leaf area (LA) and plant height (PH) (Figure S11a,c,e). The same trait combinations distinguished shade‐tolerant and shade‐intolerant angiosperm assemblages (Figure S11b,d). In contrast, shade‐tolerant gymnosperms showed lower values of SSD, SM, LA and PH compared to intolerant ones (Figure 11f). Leaf traits associated with Traits.PC1 (SLA‐LN) generally exhibited less importance than the traits associated with Traits.PC2, except for shade tolerance in evergreen gymnosperms (Figure 2f), for which higher SLA‐LN values were associated with increased shade tolerance (Figure S11f). In general, higher SLA‐LN values aligned with greater tolerance to drought in angiosperm assemblages (Figure S11a,c), although the pattern was reversed for gymnosperms (Figure S11e).

Beyond the trait dimensions, the soil dimension associated with nutrient availability (Soil.PC1) emerged as a strong predictor for the drought‐waterlogging/cold tolerance across all PFTs (Figure 2). Drought‐tolerant assemblages were consistently associated with higher soil pH values (positive side of Soil.PC1) and reduced soil nitrogen and organic matter content (Figure S11a,c,e), while waterlogging/cold‐tolerant assemblages showed a reverse pattern. In contrast, the shade tolerance assemblages exhibited varying patterns across PFTs, with the assemblages of shade‐tolerant evergreen angiosperms and gymnosperms being associated with higher and lower soil pH values, respectively (Figure S11d,f). Furthermore, we found that waterlogging/cold‐tolerant assemblages were associated with lower coarse fragment content and greater bulk density (lower values along Soil.PC3) while drought‐tolerant deciduous angiosperm assemblages (Figure 2a) showed the opposite pattern (Figure 11a).

Overall, the climatic dimensions were relatively less important as predictors of the drought‐waterlogging/cold trade‐off compared to functional traits and soil dimensions. However, the climatic dimensions emerged as prominent predictors of shade tolerance with some differences between PFTs (Figure 2). Shade‐tolerant deciduous angiosperm assemblages were associated with a higher mean annual temperature, a higher mean temperature during the driest/coldest months, a prolonged growing season and reduced seasonality (higher values along Clim.PC1) along with higher annual precipitation (lower values along Clim.PC2) and reduced precipitation seasonality (higher values along Clim.PC4). (Figure S11b). Similarly, shade‐tolerant assemblages for evergreen angiosperms and gymnosperms were associated with higher mean annual precipitation (lower values along Clim.PC2) and higher temperature and reduced seasonality (lower values along Clim.PC2), respectively (Figure S11d,f). The mean temperature during the warmest/wettest quarter and growing degree days (Clim.PC3) also played a significant role in distinguishing waterlogging/cold and drought‐tolerant assemblages for evergreen gymnosperms, with drought‐tolerant assemblages associated with a higher mean annual temperature (Figure S11c,e).

### 3.2 Stress Tolerance Biomes

**FIGURE 3:**
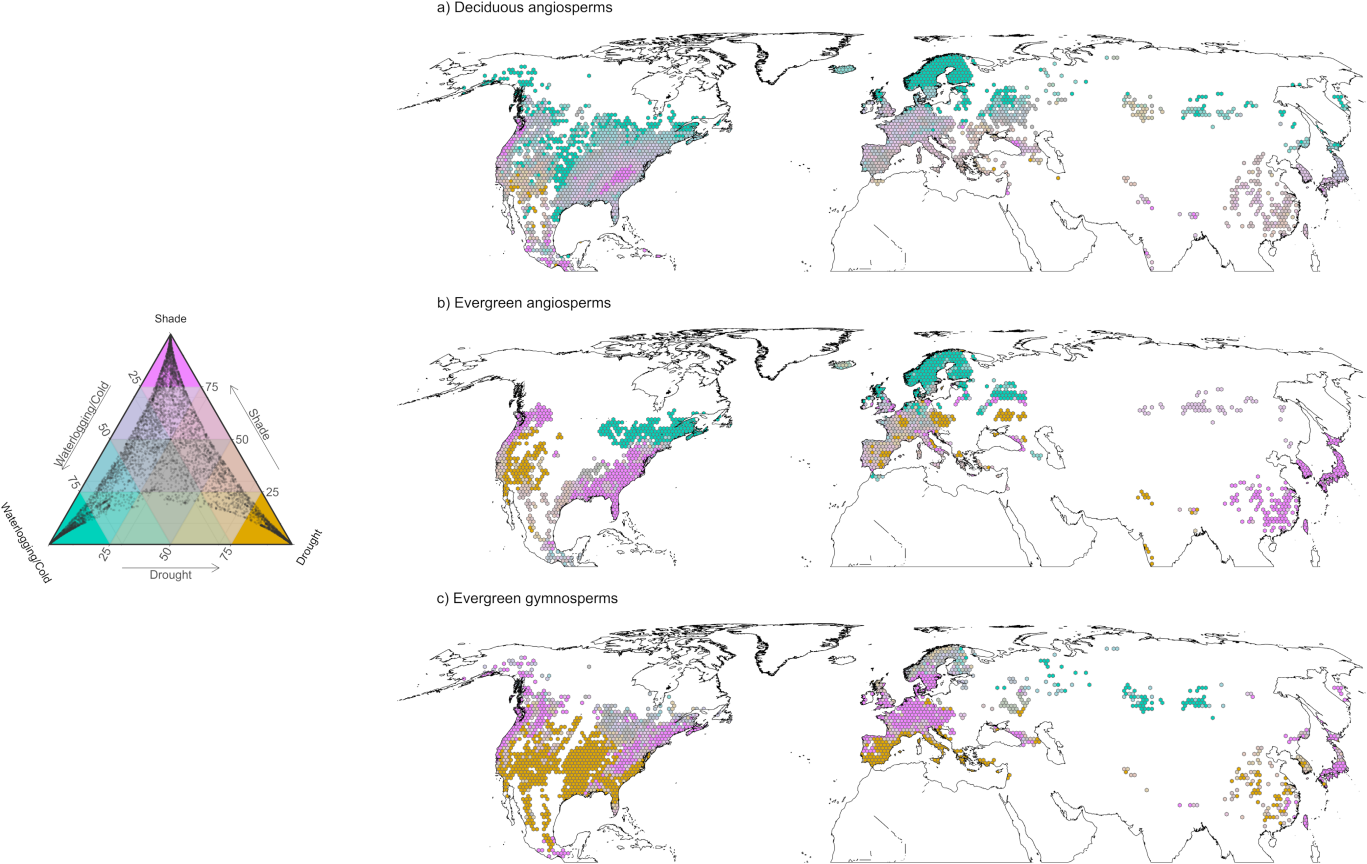
Stress Tolerance Biomes (STBs) of woody plants in the Northern Hemisphere for (a) deciduous and (b) evergreen angiosperms and (c) evergreen gymnosperms. The ternary plot on the left represents the different combinations of drought, shade and waterlogging/cold tolerance. The three STBs are highlighted, corresponding to the three end point tolerance strategies, identifying drought (dark yellow), shade (magenta) and waterlogging/cold (cyan) specialist assemblages. Black dots represent the distribution of the assemblages in relation to their degree of membership in these three end‐point strategies. Assemblages in the middle of the ternary scale are associated with low‐intermediate tolerance towards all three stressors. STBs were defined after rescaling the membership degree matrix obtained from the fuzzy—*k*‐means clustering using the RF model predictions for the waterlogging/cold drought tolerance trade‐off and the shade tolerance spectrum, considering all the hexagons (see Section 2—Characterisation of the Stress Tolerance Biomes and polytolerance hotspots section). Incomplete cover of the stress tolerance biome map reflects either missing data in GBIF (e.g., most Siberia), limited coverage of woody species tolerance data set for some species‐rich regions (East Asia) or lack of woody vegetation (North American prairie region, Arctic desert).

Our results revealed contrasting spatial distributions of the stress tolerance biomes (STBs) among PFTs (Figure 3). The number of assemblages in each STB by PFT is shown in Table S6. The ecological features of each STB are reported in Box 1.

BOX 1 **Features of the Stress Tolerance Biomes (STBs).**

Each STB presented contrasting spatial distribution patterns across PFTs and occupied distinct regions of the climatic, soil and trait space (Figures S15–S17). The most common species found in each STB are reported in Table S7, along with some representative cases of associated natural vegetation (Figure S20).

***Drought stress tolerance biome***

*North America*

The drought STB for deciduous angiosperm assemblages was primarily associated with xeric deserts and dry shrublands of southwest America, notably near the Sonoran, Mojave and Chihuahuan deserts (Figure 3a). The drought‐tolerant evergreen angiosperm assemblages in North America showed a wider distribution, encompassing Californian Mediterranean forests and grasslands, eastern xeric shrublands, eastern temperate forests, the Palouse prairie and extending to the deserts of central Mexico (Figure 3b). Gymnosperm drought‐tolerant assemblages spanned extensive regions from the eastern to the western coast, covering areas such as the southeast warm temperate forests, central savannas and grasslands, western xeric deserts and extending south to the pine‐oak forests and matorral of central Mexico (Figure 3c).

*Europe*

The drought STB for angiosperms were primarily associated with Mediterranean and temperate forests, extending to parts of the western European and Pannonian forests, the eastern European steppes and the Indomalayan subtropical dry broadleaf forests (Figure 3b). For evergreen gymnosperms, the drought STB in Europe was mainly linked to Mediterranean forests, with occurrences in the Caucasus mixed forest and Pontic steppe (Figure 3c).

*East Asia*

Angiosperm assemblages were not found within the drought STB. However, drought STB for gymnosperms was primarily associated with the subtropical and temperate broadleaf forests of eastern China (Figure 3c).

***Shade stress tolerance biome***

*North America*

The shade STB for angiosperms comprises northwest temperate conifer forests and certain south‐eastern areas, extending south to the subtropical dry broadleaf forests of Central America (Figure 3a,b). For gymnosperms, shade STB was strongly associated with Appalachian forests and temperate forests of New England and the Northeast region of Eastern North America. In contrast, in Western North America, the shade STB for gymnosperms was associated with the North‐western temperate conifer forests and Northern boreal forests (Figure 3c).

*Europe*

Shade STB for evergreen angiosperm assemblages also spread to various temperate and Mediterranean forest areas in Central Europe. These ranged from the semi‐deciduous and mixed forests in South Italy and the Po Basin to the English Lowlands beech forests, extending to the Baltic and Central European mixed forests, as well as the Caucasus mixed forests (Figure 3b). In contrast, shade STB assemblages for gymnosperm were mostly associated with the Western broadleaf and Atlantic mixed forests as well as with the Western Carpathian montane forests (Figure 3c).

*East Asia*

The shade STB for evergreen angiosperm was predominant in correspondence of subtropical and temperate broadleaf and mixed forests in eastern China and Japan (Figure 3a,b). For gymnosperms, shade STB was linked to mixed forests and in proximity of the Manchurian mixed forests, with some of these assemblages appearing further north, corresponding to the Russian Bering tundra (Figure 3c).

***Waterlogging/cold stress tolerance biome***

*North America*

The waterlogging/cold STB for deciduous angiosperms was predominantly located at higher latitudes, particularly near the limits of the boreal forest and the north‐eastern temperate forests. An exception was a substantial region in the western and central‐southern United States Central forest–grasslands transition prairies, dry shrublands and woodlands (Figure 3a,b). Evergreen angiosperm assemblages were found exclusively in the north‐eastern temperate forests, stretching to the Great Lakes forests. Conversely, no waterlogging/cold gymnosperm assemblages were identified in North America (Figure 3c).

*Europe*

For angiosperms, the waterlogging/cold STB primarily encompassed the Scandinavian Peninsula and Icelandic boreal forests, as well as the regions of the western Sarmatic mixed forests in the Baltic region and Scottish conifer forests (Figure 3a,b). For gymnosperm, these assemblages were particularly present in the Ural Mountains (Figure 3c).

*East Asia*

The waterlogging/cold STB for deciduous angiosperms was located in parts of the eastern Siberian boreal forests and the temperate deciduous forests of eastern China (Figure 3a). Evergreen angiosperms assemblages were not observed in East Asia, while for gymnosperms, the waterlogging/cold STB was found in the vicinity of the Sayan Mountains and the southern East Siberian boreal forests and dry shrublands (Figure 3b,c).

Angiosperm assemblages in drought STB were predominantly found in more xeric biomes, such as deserts (comprising 48.3% and 15% of the total deciduous and evergreen assemblages in the drought STB, respectively), grasslands‐dry shrublands (24.1% and 6.4%) and savanna‐dry woodlands (10.3% and 32.2%) (Figure S12a,b). A significant proportion of evergreen angiosperm assemblages were located in temperate forests (31.3%), while the remaining deciduous and evergreen assemblages were associated with warm temperate forests (6.9% and 9.1%, respectively) and tropical‐subtropical forests (10.3% and 5.4%, respectively). Conversely, the gymnosperm assemblages in the drought STB were mostly associated with warm‐temperate (40.2%) and temperate (16.1%) forests, followed by more xeric biomes (grasslands‐dry shrublands: 20.2%, savanna‐dry woodland: 16.5%, desert: 6.5%).

The shade STB assemblages were predominantly found in temperate forests (35.8%, 43.1% and 68% of the total assemblages for deciduous and evergreen angiosperms, and evergreen gymnosperms, respectively) and warm‐temperate forests (50.8%, 52.2% and 16%, respectively) (Figure S12a–c). Some evergreen assemblages belonged to cooler boreal forests (2.5% and 10.5% for angiosperms and gymnosperms, respectively) (Figure S12b,c), whereas a few deciduous angiosperm assemblages were associated with warmer grassland‐dry shrublands (3.4% of the total) and tropical‐subtropical forests (6.7%) (Figure S12a).

Most of the waterlogging/cold STB for angiosperm assemblages were associated with temperate forests (30% and 64.5% for deciduous and evergreen, respectively) and boreal forests (41.5% and 30%, respectively) as well as the tundra biome (6.7% and 5.1%). Additionally, some deciduous assemblages were also associated with dry woodlands (6.2% of the total) and shrublands (13.9%). In contrast, the majority of gymnosperm assemblages in the waterlogging/cold STB were linked to boreal forests (87.7%), with the remainder being mostly associated with dry shrublands (9.2%) (Figure S12c).

### 3.3 Polytolerance Hotspots

**FIGURE 4:**
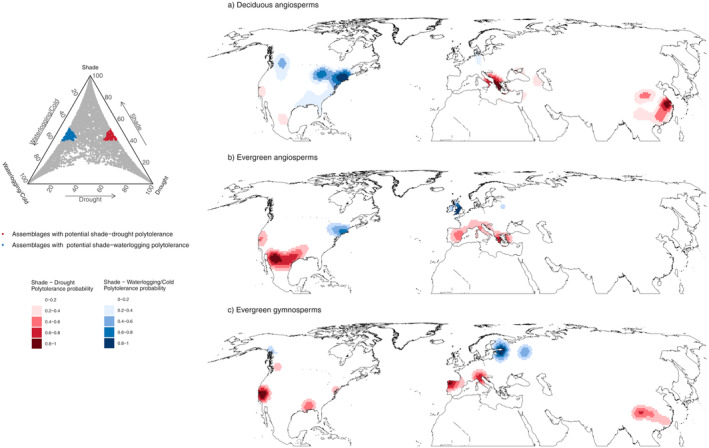
Polytolerance hotspots of woody plants in the Northern Hemisphere for (a) deciduous and (b) evergreen angiosperms and (c) evergreen gymnosperms. The ternary plot on the left represents the distribution of the assemblages (grey dots) in relation to their membership degree towards drought, shade and waterlogging/cold tolerance. Assemblages that show potential polytolerance to either shade—drought (red) or shade—waterlogging/cold are highlighted. The areas defining the polytolerance hotspots were identified using a spatial kernel density estimation on the polytolerant assemblages. Polytolerance hotspots colours reflect the probability of finding polytolerance assemblages, ranging from high (0.8–1, dark red and dark blue for shade‐drought and shade‐waterlogging/cold, respectively) to low (0.2–0.4, light red and light blue for shade‐drought and shade‐waterlogging/cold, respectively).

We identified regions exhibiting maximal shade‐drought or shade‐waterlogging/cold polytolerance, that is, polytolerance hotspots (Figure 4). These areas displayed contrasting geographical distributions among PFTs. Most of the shade‐drought polytolerant assemblages were found in evergreen angiosperms (*n* of total hexagons = 74, 3.8% of the total evergreen angiosperm assemblages), followed by deciduous angiosperms (*n* = 38, 1.4%) and evergreen gymnosperms (*n* = 26, 1.1%). Conversely, most shade‐waterlogging/cold polytolerant assemblages were found in deciduous angiosperms (*n* = 142, 5%), followed by evergreen gymnosperms (*n* = 30, 1.3%) and evergreen angiosperms (*n* = 17, 0.8%). The ecological features of each polytolerance hotspot are reported in Box 2.

BOX 2 **Features of Polytolerance Hotspots Across Continents.**

Each polytolerance hotspot presented contrasting spatial distribution patterns across PFTs and occupied distinct regions of the climatic, soil and trait space (Figures S18 and S19). The most common species found in each polytolerance hotspot are reported in Table S7, along with some representative cases of associated natural vegetation (Figure S21).

***Shade‐Drought polytolerance hotspots***

A polytolerance hotspot for angiosperm assemblages was prevalently found in the Mediterranean basin (Figure 4a,b), particularly in the Aegean and Turkish warm‐temperate Mediterranean forests, as well as in Southern Italy for deciduous angiosperms, and extending to the entire Western Mediterranean coast for evergreens. In contrast, gymnosperm assemblages exhibiting polytolerance were predominantly found in Northern Italy, near the Po basin, and in the temperate Cantabrian mixed forests of northwest Spain (Figure 4a,b). In North America, evergreen shade‐drought polytolerance hotspots were found in the hot xeric shrublands of the southwest of the United States, extending to the warm‐temperate forests of the western Gulf and the Texas shrublands. For gymnosperms, these polytolerance hotspots were identified in the California chaparral (Figure 4c). In East Asia, deciduous angiosperms polytolerant assemblages were predominantly found in the region corresponding to the Changjiang plain evergreen forests (Figure 4a), while gymnosperm polytolerance hotspots in this region were mainly in the temperate conifer forests of southeast Tibet and the Sichuan region (Figure 4c).

***Shade–Waterlogging/cold polytolerance hotspots***

In North America, polytolerance hotspots for angiosperms were primarily located in the temperate forests of the Northeast Coast and around the Great Lakes area (Figure 4a,b), with deciduous angiosperms also identifying a hotspot in the Northern Rockies temperate conifer forests. No polytolerance hotspots were detected for gymnosperms in North America. In Europe, a hotspot for evergreen angiosperms was found in correspondence of the Celtic broadleaf forests of Great Britain and Ireland (Figure 4b). On the other hand, gymnosperm hotspots for shade‐waterlogging/cold tolerance span a significant area, encompassing the north‐western portion of the Sarmatic mixed forests and extending into parts of the eastern Scandinavian and Russian taiga (Figure 4c). East Asia did not reveal polytolerance hotspots for shade waterlogging/cold tolerance.

Most shade‐drought polytolerant assemblages across all PFTs were associated with warm temperate (68.4%, 54.1% and 42.3% of the total assemblages for deciduous angiosperms, evergreen angiosperms, and evergreen gymnosperms, respectively) and temperate forests (21.1%, 8.1% and 34.6%, respectively), as well as dry shrublands (5.3%, 35.1% and 15.4%, respectively) (Figure S12d–f).

Almost all shade‐waterlogging/cold polytolerant angiosperm assemblages were associated with temperate (64.8% and 82% for deciduous and evergreen, respectively) and warm‐temperate forests (21.8% and 18%, respectively), with some deciduous assemblages also linked to dry woodland (7.7%) and boreal biomes (4.9%) (Figure S12d,e). Shade‐waterlogging/cold gymnosperm assemblages were predominantly linked to boreal (70% of the total) and temperate forest biomes (27%) (Figure S12f).

## 4 Discussion

We identified climatic, soil and functional constraints underlying the trade‐offs among drought, shade and waterlogging/cold tolerance of woody plants across the Northern Hemisphere. This information allowed us to summarise such trade‐offs in terms of three major stress tolerance biomes (STB) and two types of polytolerance hotspots, highlighting regions across the Northern Hemisphere where species assemblages converge toward specialised stress tolerance strategies. These regions are associated with highly contrasting ecological characteristics, providing the basis for explaining the trade‐offs that limit polytolerance in woody plants across the Northern Hemisphere.

### 4.1 Descriptors of Stress Tolerance Strategies

Trait syndromes and soil fertility were the main descriptors of the drought‐waterlogging/cold tolerance trade‐off in woody plants of the Northern Hemisphere (Figure 2, Figure S11a,c,e). In particular, size‐related traits exerted an overall greater effect compared to the leaf economic dimension, mirroring the patterns found by Scheiter et al. (2023) using a dynamic global vegetation model. This is because variation in size traits generally follows a latitudinal gradient (Joswig et al. 2022) and, as such, much of the predictable latitudinal variation in drought and waterlogging/cold tolerance strategies (Nogués‐Bravo et al. 2014; Puglielli, Tordoni et al. 2023) is well captured by the size‐related dimension. In contrast, leaf economic traits are more closely related to local climatic conditions, thus exerting a more nuanced effect in describing large‐scale patterns of tolerance to abiotic stress in woody plants (Xu, Tomlinson, and Li 2020; Joswig et al. 2022; Scheiter et al. 2023). The effect of soil fertility (Soil. PC1) in defining the species sorting along the drought‐waterlogging/cold tolerance trade‐off (Figure 2a,c,e) is consistent with soil properties being an overall better predictor of traits on a large scale compared to climate (Ordoñez et al. 2009; Maire et al. 2015; Simpson, Richardson, and Laughlin 2016). These results highlight the direct and indirect role of soil fertility in influencing stress tolerance strategies by shaping plant functional features. The availability of soil nutrients is crucial for carbon fixation and stress tolerance; greater availability of nutrients leads to enhanced carbon assimilation, increased potential for carbon storage and an increased capacity to withstand stress (Coskun, Britto, and Kronzucker 2016). Soil fertility is shaped by latitudinal patterns of temperature and precipitation (Huston and Wolverton 2009), thus elucidating how environmental factors and traits that covary with latitude sort assemblages along the drought‐waterlogging/cold trade‐off.

In addition to traits, climate was key to explaining the spatial distribution of shade tolerance (Figure 2, Figure S11b). In particular, a higher mean annual temperature, a longer growing season (Clim.PC1) and a higher mean annual precipitation (Clim.PC2) were always associated with greater shade tolerance in all plant functional types (PFTs) (Figure 2b,d,f). This result provides the first unequivocal evidence for the hypothesis that the duration of the growing season is critical for shade‐tolerant plants to maintain a positive carbon balance (Niinemets and Valladares 2008). Multiple studies hypothesized that a longer growing season, coupled with sufficient water availability, enables plants to withstand higher levels of shade (Valladares and Niinemets 2008; Laanisto and Niinemets 2015; Valladares et al. 2016). This is also because ecosystems with ample water and nutrient availability generally select for dense canopies, thus favouring the selection of shade tolerance strategies. In sum, shade‐tolerant assemblages preferentially occur in warm and wet regions at lower latitudes across the Northern Hemisphere (Figures S12–S14). This interpretation explains the difference between the descriptors of shade tolerance compared to the waterlogging/cold‐drought tolerance syndrome and possibly their independence in the STS.

### 4.2 Stress Tolerance Biomes (STBs) and Polytolerance Hotspots

Biome classifications have historically been used to group vegetation according to climatic conditions, species composition and physiognomy or soil features (e.g., Dinerstein et al. 2017; Beck et al. 2018; Hengl et al. 2018; Mucina 2019; Fischer, Walentowitz, and Beierkuhnlein 2022). Here, we applied the same concept to summarise the main abiotic stress tolerance syndromes in the Northern Hemisphere, as recently done for herbivory resistance syndromes (Dantas and Pausas 2022). Summarising complex patterns into a tractable one can help address the gap of how to integrate additional dimensions related to the biological characteristics of the species into macroecological analyses (Puglielli and Pärtel 2023). As such, the proposed STB classification can be easily integrated with other classification systems or already drawn macroecological patterns (e.g., species richness), especially considering that they add non‐redundant information to existing biomes classifications (Figures S12 and S13).

For these purposes, we define STBs as regions in the Northern Hemisphere where diverse assemblages have converged towards specialised stress tolerance strategies to cope with specific environmental requirements. STBs provide a representation of the three vertices of the triangular STS in geographical space (Figures 1 and 3). The emergence of the STBs is inherently explained by optimisation principles, which suggest that species are inclined to evolve specialised adaptations to optimise their performance for specific functional objectives (Marks and Lechowicz 2006; Shoval et al. 2012). In this framework, STBs delineate areas boundaries that might represent eco‐evolutionary limits to stress tolerance strategies. The assemblages in each STB have in fact converged on specialised stress tolerance strategies as determined by trade‐offs between functional attributes and environmental pressures (Reu et al. 2011; Puglielli, Hutchings, and Laanisto 2021). Finally, defining each STB by unique combinations of climatic, soil and functional characteristics (Figures S15–S17), independently of geographic location, implies that the STBs exhibit little overlap with each other, thereby providing a comprehensive picture of the functional and environmental constraints underlying trade‐offs among tolerances to different stressors in woody plants. Our analysis also allowed us to identify key regions in the Northern Hemisphere demonstrating potential for polytolerance, that is, polytolerance hotspots (Figure 4).

Polytolerance is considered rare in woody species in the Northern Hemisphere, due to biophysical constraints and functional trade‐offs (Niinemets and Valladares 2006; Valladares et al. 2008). Indeed, our findings confirm that potential polytolerance hotspots occupy a significantly smaller area compared to the larger STBs. Importantly, given that these small areas harbour unique tolerance strategies, and they are associated with specific features in terms of both environmental constraints and plant functional characteristics, any impact in these areas could result in the loss of unique functions. Our analysis revealed that shade‐drought polytolerant assemblages are predominantly found in regions with elevated temperatures, extended growing seasons, higher soil pH and lower nutrient levels, but with generally higher precipitation than those observed in the drought STB (Figures S15 and S18). These regions include areas spanning from warm‐temperate evergreen Mediterranean forests to temperate evergreen forests in East China, as well as specific biomes such as North American xeric shrublands, and chaparral (Figure 4, Box 2). The assemblages in these areas were associated with greater wood density, seed mass and plant height, suggesting a combination of conservative resource strategies coupled with a longer growing season to cope with both shade and drought stress (Niinemets and Valladares 2008). In contrast, shade‐waterlogging/cold polytolerant assemblages were mainly found in specific regions of eastern North American coastal forests and in gymnosperm assemblages in the Scandinavian and Russian boreal forests. These regions are characterised by comparatively higher precipitation, cooler temperatures and more fertile soils, which support adaptations to both shade and waterlogging/cold stresses (Niinemets and Valladares 2006; Craine et al. 2012). The lower plant height, wood density and seed mass observed in these assemblages confirmed a shift towards more acquisitive resource strategies in response to the reduced length of the growing period (Rueda, Godoy, and Hawkins 2017; Pavanetto et al. 2024), supporting the theoretical predictions of Niinemets and Valladares (2008).

## 5 Conclusions

The definition of stress tolerance biomes (STBs) and polytolerance hotspots in woody plants of the Northern Hemisphere provides an empirical macroecological framework to describe and understand the trade‐offs limiting the diversity of abiotic stress tolerance strategies in woody plants. We showed that STBs and polytolerance hotspots were associated with multiple biomes as defined by already existing classifications (Figure S13), suggesting that woody plant stress tolerance strategies cannot be inferred solely based on existing vegetation biomes.

## Author Contributions

Nicola Pavanetto and Giacomo Puglielli conceptualised the study and planned the data analysis. Nicola Pavanetto performed the data analysis and wrote the first draft of the manuscript with substantial inputs from Giacomo Puglielli, Ülo Niinemets and Marta Rueda. All the authors contributed to the critical revision of the manuscript to produce the final version.

## Conflicts of Interest

The authors declare no conflicts of interest.

### Peer Review

The peer review history for this article is available at https://www.webofscience.com/api/gateway/wos/peer‐review/10.1111/ele.70016.

## Supporting information


Data S1.

## Data Availability

The raw data sets, metadata and all the R material needed to replicate the main analysis and figure in this manuscript are publicly available in ‘Figshare’ (DOI: https://doi.org/10.6084/m9.figshare.25392295.v2).
