# Supplementary material for: Macroecology of Abiotic Stress Tolerance in Woody Plants of the Northern Hemisphere: Tolerance Biomes and Polytolerance Hotspots
Source: Ecol Lett. 2024 Dec 2;27(11):e70016. doi: 10.1111/ele.70016 (PMC11612541; doi:10.1111/ele.70016)
Supplement: Supplementary file 1 — Data S1. [file ELE-27-0-s001.docx]

**Supplementary Information**

**Note S1** GBIF occurences validation and environmental and geographical filtering

**Table S1** List of the environmental variables used in this study

**Fig. S1** Comparison between the environmental space occupied by species occurrence records before and after the environmental filtering procedure

**Table S2** Correlation matrix between hexagons abundance-weighted trait averages with environmental filtered occurrences and with the full dataset.

**Note S2** Data spatial aggregation

**Fig. S2** Distribution of the hexagon grid defined using species occurrences across the major biomes of the Northern Hemisphere

**Table S3** Most abundant species and total number of different species in each major biomes.

**Note S3** Climate, soil, and functional traits dimensions

**Table S4** Loadings of the varimax-rotated PCAs and variance explained of each principal component for the climatic, soil, and trait data

**Fig. S3** Kernel density (i.e., probability) estimation for the “climate space” defined by the varimax-rotated PCA using the mean climatic variables

**Fig. S4** Kernel density estimation (i.e., probability) for the “soil space” defined by the PCA using the mean soil variables

**Fig. S5** Kernel density estimation (i.e., probability) for the “trait space” defined by the PCA using the weighted mean functional traits variables

**Fig. S6** Correlation matrix between climate, soil and trait principal components.

**Note S4** Random forest models specification

**Table S5** Effects of longitude (lon) and latitude (lat) on the residuals extracted from the random forest models

**Fig. S7** Uncertainty map of random forest predictions for the waterlogging/cold –drought tolerance trade-off.

**Fig. S8** Uncertainty map of random forest predictions for the shade tolerance spectrum

**Fig. S9** Fuzzy k-means cluster analysis using RF models predictions of the cold/wt-drought trade-off and the shade tolerance spectrum

**Fig. S10** Spatial distribution of the assemblages after the after the Fuzzy k-means classification analysis

**Fig. S11** Marginal effects of the most important predictors on the Stress Tolerance Space (STS) axes.

**Table S6** Number of assemblages associated to each of the Stress Tolerance Biomes (STB) and polytolerance hotspots

**Fig. S12** Main Potential Natural Vegetation (PNV) biomes associated to each Stress Tolerance Biome (STB) and polytolerance hotspots

**Fig. S13** Ecoregions biomes associated to each Stress Tolerance Biome (STB) and polytolerance hotspots

**Fig. S14** Abiotic stress tolerance strategies in the geographic space

**Fig. S15** The drought Stress Tolerance Biome inside the climate, soil and traits spaces

**Fig. S16** The shade Stress Tolerance Biome inside the climate, soil and traits spaces

**Fig. S17** The waterlogging/cold Stress Tolerance Biome inside the climate, soil and traits spaces

**Fig. S18** The shade-drought polytolerance hotspot inside the climate, soil and traits spaces

**Fig. S19** The shade- waterlogging/cold polytolerance hotspot inside the climate, soil and traits spaces

**Table S7** Most abundant species in each potential natural vegetation biomes associated with each Stress Tolerance Biomes and polytolerance hotspot.

**Fig. S20** Representative cases of natural vegetation for each stress tolerance biomes (STB).

**Fig. S21** Representative cases of natural vegetation for each polytolerance hotspot.

**Note S1. GBIF occurrences validation and environmental and geographical filtering**

The density of occurrence records collected from GBIF sources exhibited significant variation across geographical regions, with certain areas having a considerably higher number of records compared to others. This discrepancy in sampling intensity has the potential to introduce biases and result in redundant and noisy information during statistical modeling (Castellanos et al., 2019). Furthermore, although GBIF has an irreplaceable value for analyses involving the use of distribution data at all scales, this database has been constructed with a large community effort as well as using automated data-filling procedures and it is highly inclusive, thereby requiring carefulness when using such data (see Maldonado et al., 2015; Zizka et al., 2020 for a detailed account of main GBIF limitations and how to overcome them). As a first step, we used the R package CoordinateCleaner (Zizka et al., 2021) to flag observations with problematic coordinates (e.g., coordinate coinciding with state capitals or nearby scientific institutions that can introduce notable sampling bias). We also removed duplicate observations, observations wrongly assigned to country regions, which suggests inaccurate sampling precision, and outliers. Outliers were identified as observations where the average distance from all other records of the same species exceeded at least five times the interquartile range of the mean distance for all records of that particular species.

Subsequently, we manually filtered out all the observation with coordinate uncertainty > 1000 m and observations from unsuitable (i.e., from scholarly publications and machine derived observations) or unknown data sources. Finally, we also removed old records (before 1960) and records with high individual counts (> 99,i.e., the number of individuals of the same species in the same site reported at the time of the record), as they have been repeatedly indicated as an indicator of incorrect sampling or data entry problems (Zizka et al., 2020). All species occurrence data were then subjected to an additional environmental filtering process to mitigate potential biases arising from uneven distribution of occurrences within the environmental space derived from species’ occurrences (Castellanos, 2019).

**Environmental and geographical filtering of species occurrence data**

To mitigate the impact of sampling bias on species records, we implemented an environmental filtering procedure (Varela et al., 2014; Castellanos et al., 2019). For each species, we plotted occurrence records in the multidimensional environmental space defined by a Principal Component Analysis (PCA) using the standardized climatic variables. Subsequently, following Varela et al. (2014) and Castellanos et al. (2019), we divided the environmental space into a grid of 100 equal bins, randomly selecting one occurrence record from each bin. We then utilized the 'funspace' function from the 'funspace' R package (Carmona et al., 2024) to analyze the structure of the environmental space in both the complete set of occurrences and the subset obtained through environmental filtering. ‘funspace’ estimates the probability of occurrence of trait combinations within the space defined by pairs of principal components using kernel density estimation with unconstrained bandwidth selectors by combining the functionalities available in the R packages ks (Duong et al., 2022) and TPD (Carmona, 2019). This filtering procedure favors a more balanced coverage of a species occurrence within the whole species environmental space (**Fig. S1**), accounting for potential sampling bias while still considering the natural drivers of abundance (**Table S2**).

Additionally, to further account for occurrences at range margin after the environmental filtering, that can be identified as potential outliers, we utilized the 'st_kde' function from the 'eks' R package (Duong, 2023) to estimate the (spatial) kernel density for each species using the geographical coordinates of the occurrence records. We removed all the records falling outside the 90th quantile of the multivariate probability distribution. Following the environmental and kernel density filtering steps, the final occurrence data included 884222 observations for 764 species in the STS.

**References**

Carmona, C.P., 2019. TPD: Methods for Measuring Functional Diversity Based on Trait Probability Density.

Carmona, C.P., Pavanetto, N., Puglielli, G., 2024. funspace: An R package to build, analyse and plot functional trait spaces. Diversity and Distributions n/a, e13820. https://doi.org/10.1111/ddi.13820

Castellanos, A.A., Huntley, J.W., Voelker, G., Lawing, A.M., 2019. Environmental filtering improves ecological niche models across multiple scales. Methods in Ecology and Evolution 10, 481–492. https://doi.org/10.1111/2041-210X.13142

Duong, T., 2023. eks: Tidy and Geospatial Kernel Smoothing.

Duong, T., Wand, M., Chacon, J., Gramacki, A., 2022. ks: Kernel Smoothing.

Maldonado, C., Molina, C.I., Zizka, A., Persson, C., Taylor, C.M., Albán, J., … Antonelli, A., 2015. Estimating species diversity and distribution in the era of Big Data: to what extent can we trust public databases? Global Ecology and Biogeography 24, 973–984. https://doi.org/10.1111/geb.12326

Varela, S., Anderson, R.P., García-Valdés, R., Fernández-González, F., 2014. Environmental filters reduce the effects of sampling bias and improve predictions of ecological niche models. Ecography 37, 1084–1091. https://doi.org/10.1111/j.1600-0587.2013.00441.x

Zizka, A., Antunes Carvalho, F., Calvente, A., Rocio Baez-Lizarazo, M., Cabral, A., ... Antonelli, A., 2020. No one-size-fits-all solution to clean GBIF. PeerJ 8, e9916. https://doi.org/10.7717/peerj.9916

Zizka, A., Silvestro, D., Andermann, T., Azevedo, J., Ritter, C.D., Edler, D., ... Antonelli, A., ropensci, I.S., 2021. CoordinateCleaner: Automated Cleaning of Occurrence Records from Biological Collections.

**Table S1.** List of all the environmental variables used in this study

| **Abbreviation** | **Variable** | **Type** | **Units^*^** | **Source** |
| --- | --- | --- | --- | --- |
| BIO1 | Mean annual air temperature | Climate | °C | CHELSA |
| BIO2 | Mean diurnal air temperature range | Climate | °C | CHELSA |
| BIO3 | Temperature isothermality | Climate | °C | CHELSA |
| BIO4 | Temperature seasonality | Climate | °C | CHELSA |
| BIO5 | Temperature of the warmest month | Climate | °C | CHELSA |
| BIO6 | Mean daily minimum air temperature of the coldest month | Climate | °C | CHELSA |
| BIO7 | Annual range of air temperature | Climate | °C | CHELSA |
| BIO8 | Mean daily mean air temperatures of the wettest quarter | Climate | °C | CHELSA |
| BIO9 | Mean daily mean air temperatures of the driest quarter | Climate | °C | CHELSA |
| BIO10 | Mean daily mean air temperatures of the warmest quarter | Climate | °C | CHELSA |
| BIO11 | Mean daily mean air temperatures of the coldest quarter | Climate | °C | CHELSA |
| BIO12 | Annual precipitation amount | Climate | kg m^-2^ yr^-1^ | CHELSA |
| BIO13 | Precipitation amount of the wettest month | Climate | kg m^-2^ month^-1^ | CHELSA |
| BIO14 | Precipitation amount of the driest month | Climate | kg m^-2^ month^-1^ | CHELSA |
| BIO15 | Precipitation seasonality | Climate | kg m^-2^ | CHELSA |
| BIO16 | Mean monthly precipitation amount of the wettest quarter | Climate | kg m^-2^ month^-1^ | CHELSA |
| BIO17 | Mean monthly precipitation amount of the driest quarter | Climate | kg m^-2^ month^-1^ | CHELSA |
| BIO18 | Mean monthly precipitation amount of the warmest quarter | Climate | kg m^-2^ month^-1^ | CHELSA |
| BIO19 | Mean monthly precipitation amount of the coldest quarter | Climate | kg m^-2^ month^-1^ | CHELSA |
| Gdd5 | Growing degree days heat sum above 5 | Climate | °C | CHELSA |
| Gsl | growing season length | Climate | n° of days | CHELSA |
| Scd | Snow cover days | Climate | n° of days | CHELSA |
| Clay | Clay mass fraction | Soil | % | SoilGrid |
| Silt | Silt mass fraction | Soil | % | SoilGrid |
| Sand | Sand mass fraction | Soil | % | SoilGrid |
| Coarse.frag | Coarse fragment | Soil | % | SoilGrid |
| Bulk.dens | Bulk density of the fine earth fraction | Soil | kg cm^-3^ | SoilGrid |
| Soil.nitrogen | Soil nitrogen content | Soil | g kg^-1^ | SoilGrid |
| Org.carbon | Soil organic carbon content | Soil | g kg^-1^ | SoilGrid |
| Soil.pH | Soil pH | Soil | - | SoilGrid |
| Elev | Digital Elevation Model | Topography | - | CHELSA |
| TRI | Topographic Roughness Index | Topography | - | ENVIREM |
| TopoWet | Topographic Wetness Index | Topography | - | ENVIREM |

***** Measurement units are reported as per the main source


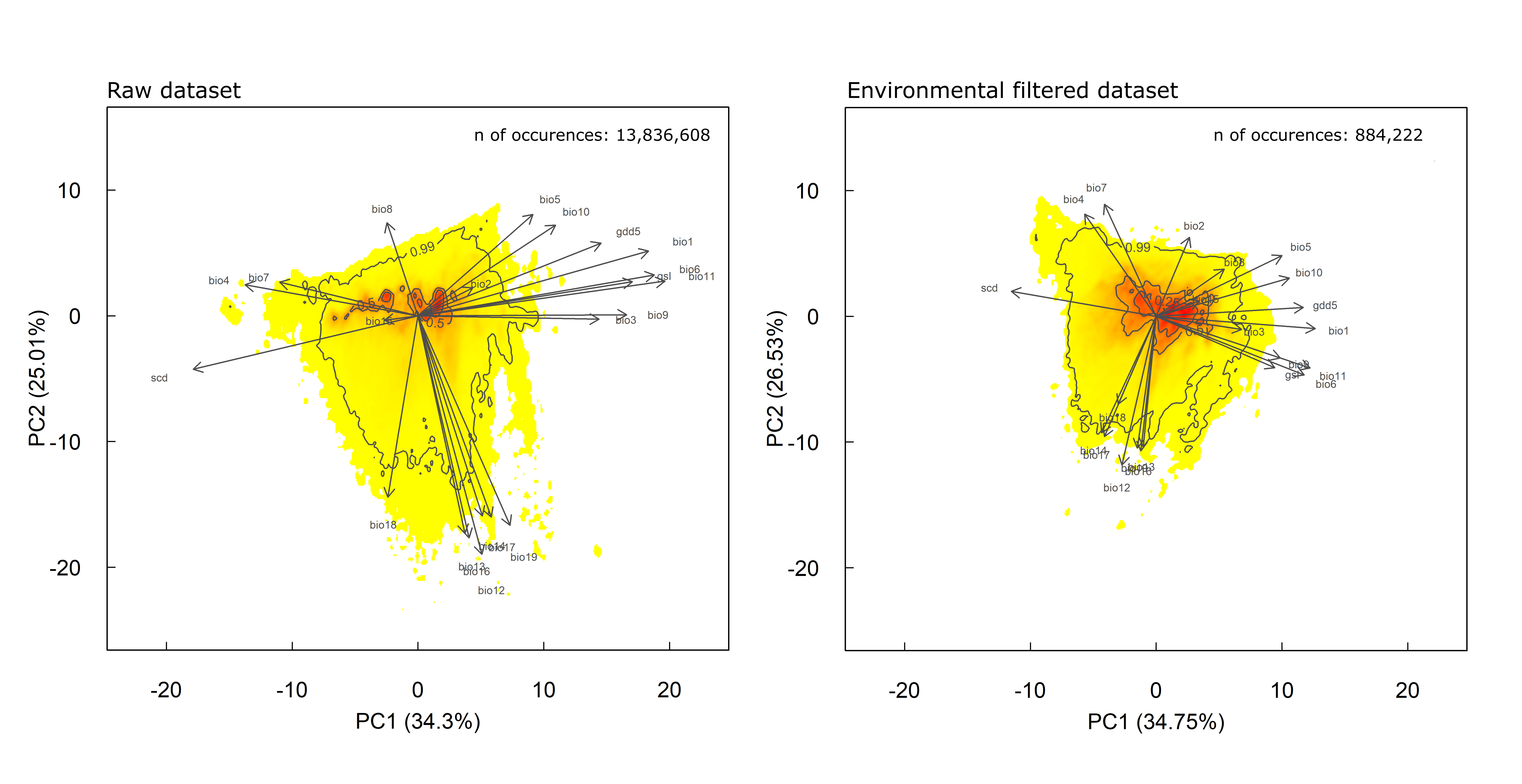


**Fig S1.** Comparison between the environmental space occupied by species occurrence records before and after the environmental filtering procedure (described in the Methods section). The environmental space was defined by conducting a Principal Component Analysis (PCA) using the CHELSA climatic variables (Table S1, Bio1-Bio19). The colors indicate the probabilistic distribution of data points, ranging from high (red) to low (light yellow) probability. Contour lines indicate the 0.99, 0.50 and 0.25 quantile of the multivariate probability distribution.

**Table S2.** Correlation indices (pearson’s *r*) between hexagon’s abundance-weighted trait averages obtained with environmental filtered occurrences (EnvFilt) and with the full dataset (Full).

|  | LA_EnvFilt_ | LN_EnvFilt_ | PH_EnvFilt_ | SLA_EnvFilt_ | SSD_EnvFilt_ | SM_EnvFilt_ |
| --- | --- | --- | --- | --- | --- | --- |
| LA_Full_ | **0.90** | 0.05 | -0.02 | 0.03 | 0.11 | 0.06 |
| LN_Full_ | 0.06 | **0.94** | -0.41 | 0.80 | 0.23 | 0.22 |
| PH_Full_ | -0.02 | -0.40 | **0.92** | -0.45 | -0.15 | 0.00 |
| SLA_Full_ | 0.04 | 0.79 | -0.44 | **0.95** | 0.18 | 0.23 |
| SSD_Full_ | 0.14 | 0.24 | -0.13 | 0.21 | **0.85** | 0.37 |
| SM_Full_ | 0.10 | 0.25 | 0.01 | 0.28 | 0.37 | **0.82** |

Trait abbreviation: sla = specific leaf area; ln = leaf nitrogen content per dry mass, la = leaf area, ssd = specific stem density, sm = seed mass, ph = plant height

**Note S2. Data spatial aggregation**

To identify the drivers of stress tolerance strategies, specifically the wt/cold-drought trade-off axis and the shade tolerance spectrum (i.e., the STS axis), and to evaluate their spatial distribution, across the Northern hemisphere we used the following approach:

1. We defined a grid of the Northern hemisphere with hexagons measuring 7500 km² each. The hexagon grid was generated using the 'st_make_grid' function from the R package 'sf' and employed an equal-area projected coordinate system (Equal Earth, EPSG:8857) to ensure consistent hexagon area across different latitudes.
2. For each hexagon, we calculated the mean value of each environmental variable by extracting the cell values from the raster layers that are covered by the hexagons. This calculation was performed using the 'exact_extract' function from the 'exactextractr' R package (Baston et al., 2022).
3. Differently from climate and edaphic conditions, the functional traits and STS axis values for each hexagon were calculated using an approach similar to community-weighted means (Lavorel et al., 2008). In particular, we weighed the traits and STS axis values based on the species relative abundances obtained from species occurrence data within each hexagon. The calculation of the weighed functional traits and STS axis values was performed separately for each plant functional type (PFTs) due to their distinct geographical distributions across the Northern hemisphere and their different trait adaptations associated with given abiotic stress tolerance strategies in the STS (Pavanetto et al., 2023). Finally, to ensure the most reliable calculation of the weighted mean and trait axis values we excluded the hexagons that included fewer than 10 total occurrences across all species per each PFTs.

**References**

Baston, D., 2022. exactextractr: Fast Extraction from Raster Datasets using Polygons.

Dinerstein, E., Olson, D., Joshi, A., Vynne, C., Burgess, N.D., Wikramanayake E. … Saleem, M., 2017. An Ecoregion-Based Approach to Protecting Half the Terrestrial Realm. BioScience 67, 534–545. https://doi.org/10.1093/biosci/bix014

Lavorel, S., Grigulis, K., McIntyre, S., Williams, N.S.G., Garden, D., Dorrough, J. … Bonis, A., 2008. Assessing functional diversity in the field – methodology matters! Functional Ecology 22, 134–147. https://doi.org/10.1111/j.1365-2435.2007.01339.x

Pavanetto, N., Carmona, C.P., Laanisto, L., Niinemets, Ü., Puglielli, G., 2024. Trait dimensions of abiotic stress tolerance in woody plants of the Northern Hemisphere. Global Ecology and Biogeography 33, 272–285. https://doi.org/10.1111/geb.13788


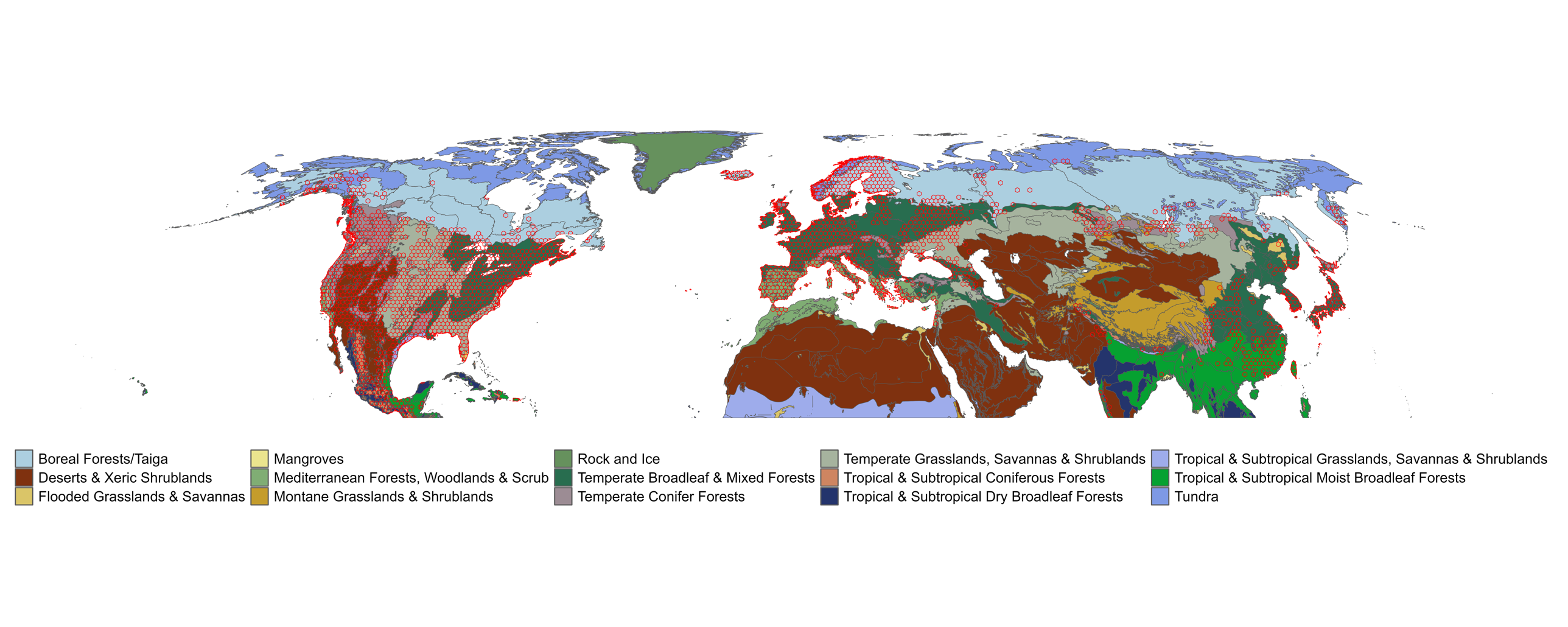


**Fig S2.** Distribution of the hexagon grid defined using species occurrences (see Methods) across the major biomes of the northern hemisphere. Each hexagon is 7500 km^2^. Biome classification follows Dinerstein et al. (2017)

**Table S3.** Most abundant species and total number of different species in each major biomes.

| Biomes | Species | N of different species |
| --- | --- | --- |
| Boreal forest/Taiga | Salix phylicifolia, Salix lanata, Vaccinium microcarpum, Salix myrtilloides, Chamaedaphne calyculata, Salix lapponum, Empetrum nigrum subsp. Hermaphroditum, Linnaea borealis, Ribes spicatum, Salix myrsinifolia, Salix starkeana, Betula nana, Rosa majalis, Daphne mezereum, Salix aurita | 228 |
| Deserts & Xeric Shrublands | Yucca brevifolia, Chilopsis linearis, Pinus monophylla,Populus fremontii, Pinus edulis, Juniperus osteosperma, Quercus turbinella, Amelanchier utahensis, Fraxinus anomala, Quercus gambelii, Celtis laevigata, Fraxinus velutina, Juniperus deppeana, Quercus emoryi, Cercoc arpus ledifolium | 159 |
| Flooded Grasslands & Savannas | Sabal palmetto, Pinus elliottii, Sideroxylon reclinatum, Ilex cassine, Quercus virginiana, Swietenia mahagoni, Salix caroliniana, Cephalanthus occidentalis, Magnolia virginiana, Morella cerifera, Fraxinus caroliniana, Rhus copallinum, Corylus heterophylla, Acer rubrum, Quercus laurifolia | 62 |
| Mangroves | Sabal palmetto, Sideroxylon celastrinum, Swietenia mahagoni, Pinus elliottii, Quercus virginiana, Ilex cassine, Prosopis juliflora, Juniperus virginiana var. Silicicola, Morella cerifera, Hibiscus rosa-sinensis, Ilex vomitoria, Juniperus virginiana, Persea borbonia, Quercus myrtifolia, Rhus copallinum | 43 |
| Mediterranean Forests, Woodlands & Scrub | Arbutus unedo, Pinus halepensis, Eriobotrya japonica, Quercus ilex, Helianthemum apenninum, Heteromeles arbutifolia, Quercus agrifolia, Cupressus sempervirens, Fraxinus ornus, Acer monspessulanum, Amelanchier ovalis, Cydonia oblonga, Quercus pubescens, Sorbus domestica, Populus nigra | 309 |
| Montane Grasslands & Shrublands | Pinus sibirica, Picea obovata, Caragana arborescens, Pinus wallichian, Cedrus deodara, Rosa acicularis, Morus alba, Rhododendron dauricum, Lycium barbarum, Tamarix ramosissima, Prunus tomentosa, Populus suaveolens, Prunus persica, Abies sibirica, Lycium chinense | 39 |
| Temperate Broadleaf & Mixed Forests | Tsuga Canadensis, Acer pensylvanicum, Rosa arvensis, Ilex aquifolium, Betula alleghaniensis, Ulex europaeus, Prunus spinose, Populus grandidentata, Rhododendron maximum, Carpinus betulus, Erica cinerea, Kalmia latifolia, Salix cinerea, Prunus laurocerasus, Ulex gallii | 644 |
| Temperate Conifer Forests | Rhododendron ferrugineum, Pseudotsuga menziesii, Thuja plicata, Alnus alnobetula, Clematis alpine, Acer macrophyllum, Helianthemum nummularium, Gaultheria shallon, Pinus ponderosa, Daphne mezereum, Rhododendron hirsutum, Rubus spectabilis, Polygala chamaebuxus, Arbutus menziesii, Pinus contorta | 660 |
| Temperate Grasslands, Savannas & Shrublands | Populus deltoids, Rhus copallinum, Cephalanthus occidentalis, Pinus palustris, Cercis Canadensis, Juniperus virginiana, Platanus occidentalis, Maclura pomifera, Rhus glabra, Diospyros virginiana, Magnolia virginiana, Sabal palmetto, Gleditsia triacanthos, Quercus macrocarpa, Salix nigra | 395 |
| Tropical & Subtropical Coniferous Forests | Juniperus deppeana, Prunus serotina, Salix discolor, Pinus strobiformis, Tilia americana, Ostrya virginiana, Carpinus caroliniana, Ptelea trifoliata, Liquidambar styraciflua, Quercus emoryi, Morella cerifera, Pseudotsuga menziesii, Cercis canadensis, Cupressus arizonica, Populus tremuloides | 82 |
| Tropical & Subtropical Dry Broadleaf Forests | Hibiscus rosa-sinensis, Prosopis juliflora, Sideroxylon celastrinum, Ptelea trifoliata, Lagerstroemia indica, Prunus serotine, Juglans major, Liquidambar styraciflua, Tilia americana, Celtis laevigata, Ostrya virginiana, Swietenia mahagoni, Carpinus caroliniana, Salix discolor, Carya illinoinensis | 32 |
| Tropical & Subtropical Grasslands, Savannas & Shrublands | Ilex vomitoria, Celtis laevigata, Quercus virginiana, Quercus nigra, Salix nigra, Zanthoxylum clava-herculis, Ulmus crassifolia, Carya illinoinensis, Ilex decidua, Platanus occidentalis, Morella cerifera, Pinus taeda, Prunus caroliniana, Cephalanthus occidentalis, Ulmus americana | 86 |
| Tropical & Subtropical Moist Broadleaf Forests | Hibiscus rosa-sinensis, Quercus glauca, Machilus japonica, Mallotus japonicas, Gardenia jasminoides, Machilus thunbergii, Cinnamomum camphora, Liquidambar styraciflua, Melia azedarach, Ardisia sieboldii, Cleyera japonica, Clerodendrum trichotomum, Symplocos paniculata, Lagerstroemia indica, Morella cerifera | 124 |
| Tundra | Empetrum nigrum subsp. Hermaphroditum, Salix herbacea, Salix lapponum, Betula nana, Vaccinium vitis-idaea, Salix phylicifolia, Empetrum nigrum, Betula pubescens, Salix lanata, Salix myrsinites, Vaccinium uliginosum, Linnaea borealis, Salix hastate, Vaccinium myrtillus, Juniperus communis | 155 |

**Note S3. Climate, soil, and functional traits dimensions**

The PCA based on climatic data revealed four main axes of variation (hereafter Clim.PC1, Clim.PC2, Clim.PC3 and Clim.PC4), accounting for a cumulative 85.7% of the total variance (**Fig. S3**). The first and third components delineated dimensions associated with temperature variables. Specifically, the first component correlated with annual temperature variation, seasonality, growing season length, and the mean temperature during the coldest/driest quarter. In contrast, the third component was related to mean temperatures during the warmest/wettest quarter and growing degree days. The second component was related to annual precipitation and mean precipitation during the wettest/warmest quarter (with higher precipitations associated with negative axis values), while the fourth component was predominantly associated with mean precipitation during the driest quarter and precipitation seasonality (**Table S4**).

Exploring the space defined by soil variables unveiled two principal axes of variation, capturing 83.33% of the total variation (**Fig. S4**). The first component (hereafter Soil.PC1) reflected a trade-off between soil pH vs. nitrogen and carbon organic content. Meanwhile the second component (hereafter Soil.PC2) represented a gradient in soil texture, and the third component was primarily associated to coarse fragments content (**Table S4**). Notably, hexagons associated with different PFTs occupied similar areas within the environmental space in both climatic and soil PCA (**Fig. S3-S4**).

In the functional trait space, we identified two main axes of variation, which accounted for 71.55% of the total variance (**Fig. S5**). The varimax-rotated first component (hereafter Trait.PC1) showed a positive association with leaf nitrogen (LN) and specific leaf area (SLA). In contrast the second component (hereafter Trait.PC2) exhibited a positive association with specific stem density (SSD) and seed mass (SM), Plant height (PH) and leaf area (LA) displayed an intermediate level of correlation between Trait.PC1 and Trait.PC2, with LA being positively associated to both axes, and PH being positively associated to the Trait.PC2 and negatively with Trait.PC1 (**Table S4**). Plant functional types (PFTs) occupied distinct regions of the trait space, particularly along the first principal component (**Fig. S5**). The climate, soil, and traits components demonstrated relatively low correlations, with the maximum Pearson’s correlation coefficients recorded at 0.54 (observed between Clim.PC3 and Soil.PC1) and 0.52 (observed between Clim.PC3 and Soil.PC3) (**Fig. S7**).

**Table S4.** Loadings of the varimax-rotated PCAs and variance explained of each principal component for the climatic, soil, and trait data. Number of dimensions (i.e. PCA Axes) retained for each PCA were estimated by Horn’s Parallel Analysis.

| **Climate** | | | | |
| --- | --- | --- | --- | --- |
|  | Loadings | | | |
| **Variable** | **PC1 _(39.11%)_** | **PC2 _(26.1%)_** | **PC3 _(11.35%)_** | **PC4 _(9.2%)_** |
| (BIO1) Mean annual air temperature | 0.72 | -0.07 | 0.67 | -0.03 |
| (BIO2) Mean diurnal air temperature range | -0.02 | 0.27 | 0.35 | -0.56 |
| (BIO3) Temperature isothermality | 0.68 | -0.01 | 0.17 | -0.46 |
| (BIO4) Temperature seasonality | -0.93 | 0.19 | 0.12 | -0.03 |
| (BIO5) Temperature of the warmest month | 0.29 | 0.16 | 0.85 | -0.21 |
| (BIO6) Mean daily minimum air temperature of the coldest month | 0.91 | -010 | 0.35 | 0.05 |
| (BIO7) Annual range of air temperature | -0.85 | 0.23 | 0.19 | -0.20 |
| (BIO8) Mean daily mean air temperatures of the wettest quarter | -0.07 | -0.14 | 0.81 | -0.15 |
| (BIO9) Mean daily mean air temperatures of the driest quarter | 0.88 | 0.05 | 0.21 | 0.01 |
| (BIO10) Mean daily mean air temperatures of the warmest quarter | 0.35 | 0.04 | 0.90 | -0.06 |
| (BIO11) Mean daily mean air temperatures of the coldest quarter | 0.89 | -0.11 | 0.42 | -0.01 |
| (BIO12) Annual precipitation amount | 0.18 | -0.86 | -0.07 | 0.42 |
| (BIO13) Precipitation amount of the wettest month | 0.19 | -0.96 | -0.01 | 0.01 |
| (BIO14) Precipitation amount of the driest month | 0.03 | -0.36 | -0.09 | 0.85 |
| (BIO15) Precipitation seasonality | 0.08 | -0.35 | 0.09 | -0.86 |
| (BIO16) Mean monthly precipitation amount of the wettest quarter | 0.20 | -0.96 | -0.03 | 0.05 |
| (BIO17) Mean monthly precipitation amount of the driest quarter | 0.05 | -0.40 | -0.07 | 0.85 |
| (BIO18) Mean monthly precipitation amount of the warmest quarter | -0.17 | -0.84 | 0.24 | 0.19 |
| (BIO19) Mean monthly precipitation amount of the coldest quarter | 0.36 | -0.44 | -0.38 | 0.44 |
| (GDD5) Growing degree days heat sum above 5 | 0.57 | -0.12 | 0.72 | -0.14 |
| (GSL) Growing season length | 0.55 | -0.26 | 0.32 | 0.42 |
| (SCD) Snow cover days | -0.75 | -0.01 | -0.54 | -0.01 |
| **Soil** | | | | |
|  | Loadings | | | |
| **Variable** | **PC1_(48.21%)_** | **PC2_(20.55%)_** | **PC3_(14.54%)_** |  |
| (CLAY) Clay mass fraction | 0.56 | -0.61 | 0.11 |  |
| (SILT) Silt mass fraction | -0.05 | -0.92 | 0.03 |  |
| (SAND) Sand mass fraction | -0.27 | 0.95 | -0.08 |  |
| (COARSE.FRAG.) Coarse fragment | -0.03 | 0.09 | -0.96 |  |
| (BULK.DENS) Bulk density of the fine earth fraction | 0.78 | -0.06 | 0.50 |  |
| (SOIL.NITROGEN) Soil nitrogen content | -0.84 | 0.21 | -0.03 |  |
| (ORG.CARBON) Soil organic carbon content | -0.88 | 0.15 | -0.20 |  |
| (SOIL.PH) Soil pH | 0.78 | -0.01 | -0.23 |  |
| **Traits** | | | | |
|  | Loadings | | | |
| **Variable** | **PC1_(46.27%)_** | **PC2_(25.28%)_** |  |  |
| (SLA) Specific Leaf Are | 0.88 | 0.20 |  |  |
| (LN) Leaf Nitrogen Content | 0.84 | 0.31 |  |  |
| (LA) Leaf Area | 0.48 | 0.65 |  |  |
| (SSD) Specific Stem Density | 0.16 | 0.72 |  |  |
| (SM) Seed Mass | 0.13 | 0.84 |  |  |
| (PH) Plant Height | -0.60 | 0.58 |  |  |


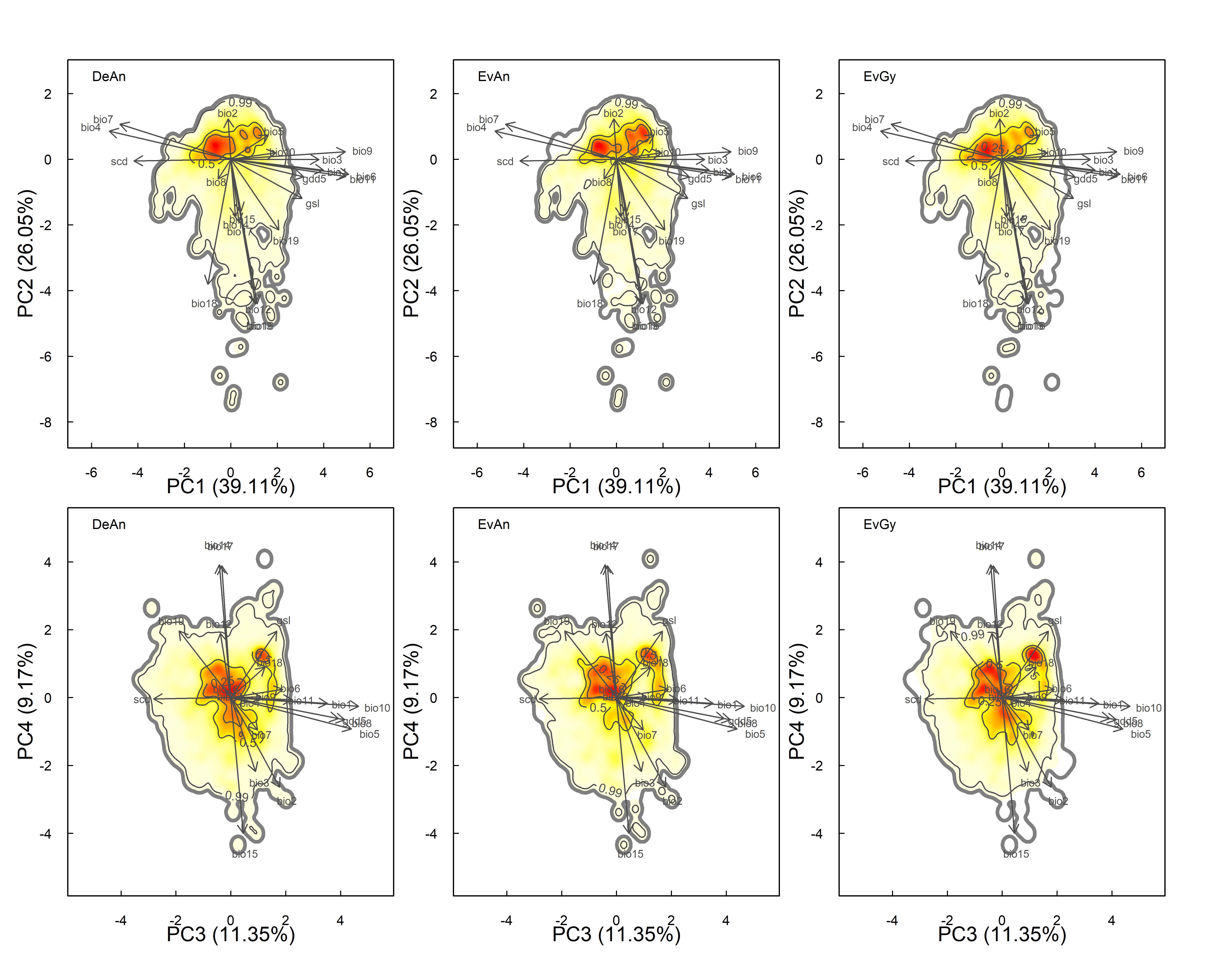


**Fig. S3** Kernel density (i.e., probability) estimation for the “climate space” defined by the varimax-rotated PCA using the mean climatic variables calculated for each hexagon polygon, for deciduous (DeAn) and evergreen (EvAn) angiosperms and evergreen gymnosperms (EvGy). The colors indicate the probabilistic distribution, ranging from high (red) to low (light yellow) probability. Contour lines indicate the 0.99, 0.50 and 0.25 quantile of the total probability distribution. For reference of the abbreviations used for climatic variables see Table S1.

**Fig. S4**. Kernel density estimation (i.e., probability) for the “soil space” defined by the PCA using the mean soil variables calculated for each hexagon polygon, for deciduous (DeAn) and evergreen (EvAn) angiosperms and evergreen gymnosperms (EvGy). The colors characterize the probabilistic distribution, ranging from high (red) to low (light yellow) probability. Contour lines indicate the 0.99, 0.50 and 0.25 quantile of the total probability distribution. For reference of the abbreviations used for soil variables see Table S1.


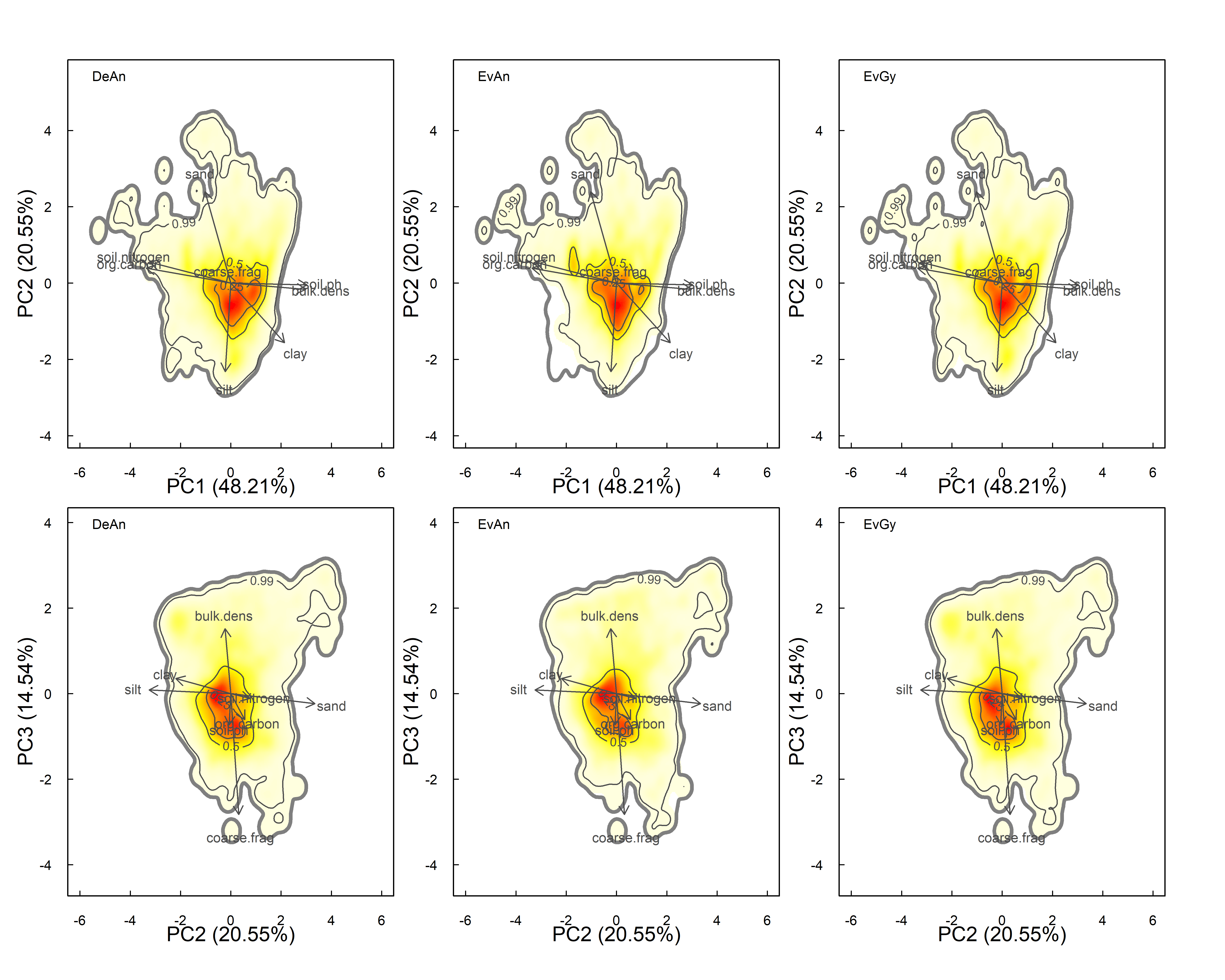

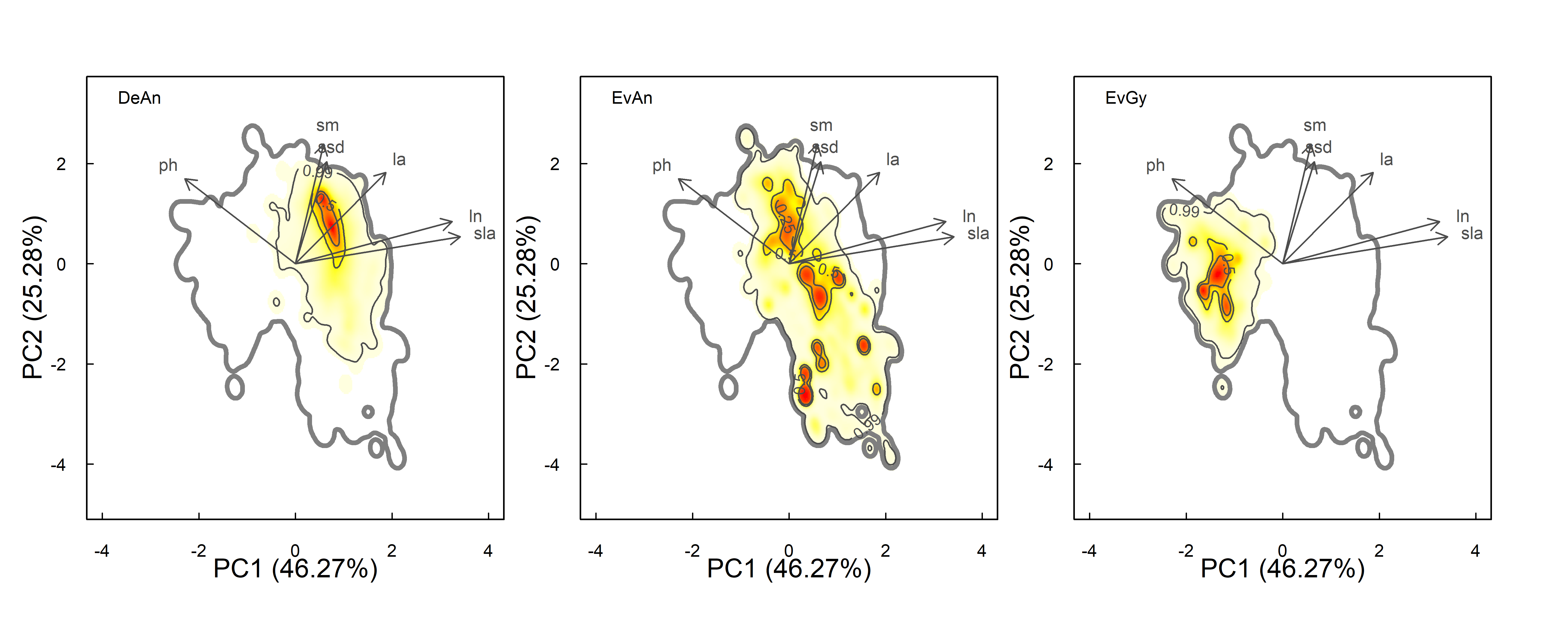


**Fig. S5.** Kernel density estimation (i.e., probability) for the “trait space” defined by the PCA using the weighted mean traits variables calculated for each hexagon polygon (see Methods for details), for deciduous (DeAn) and evergreen (EvAn) angiosperms and evergreen gymnosperms (EvGy). The colors indicate the probabilistic distribution, ranging from high (red) to low (light yellow) probability. Contour lines indicate the 0.99, 0.50 and 0.25 quantile of the total probability distribution. sla = specific leaf area; ln = leaf nitrogen content per dry mass, la = leaf area, ssd = specific stem density, sm = seed mass, ph = plant height.


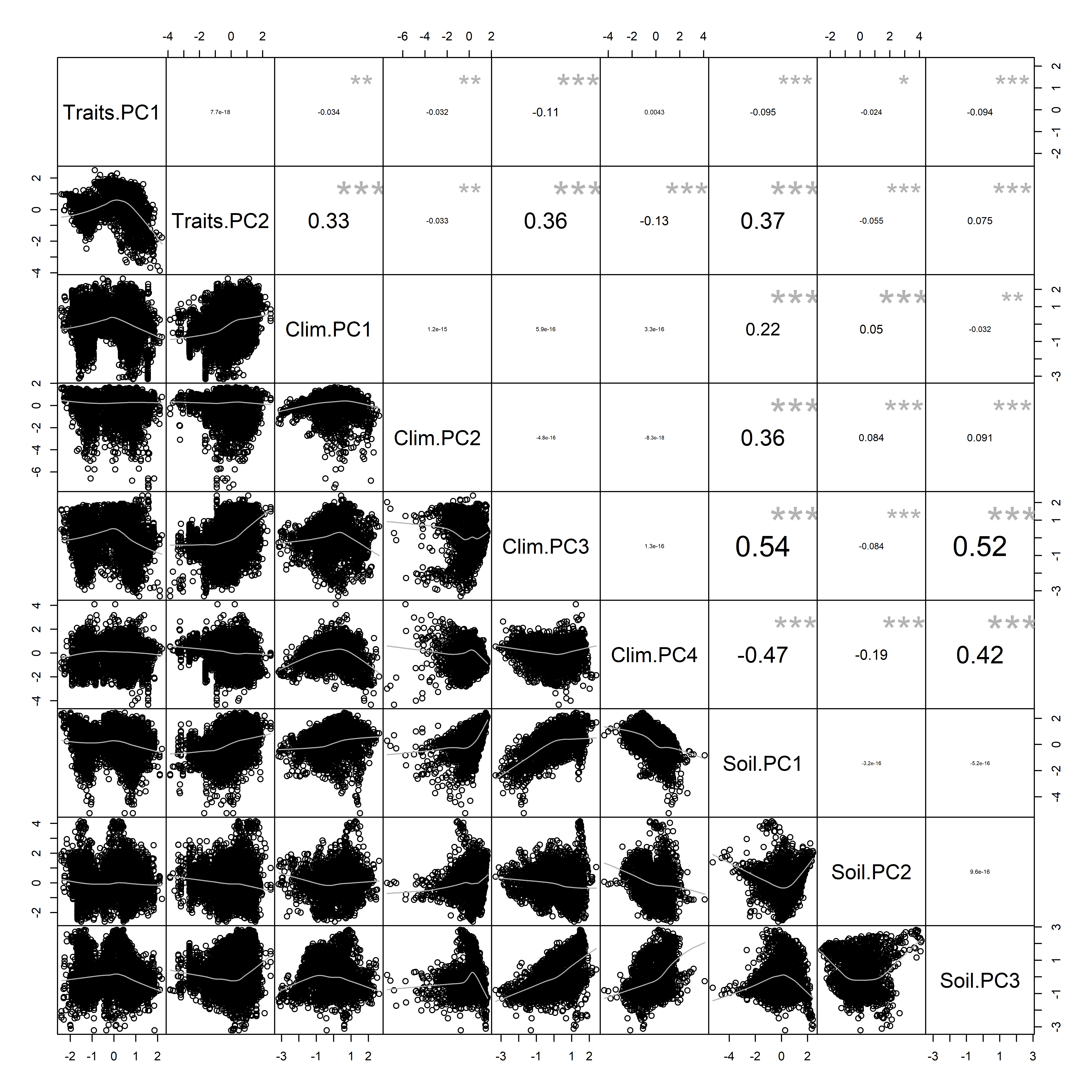


**Fig. S6. Correlation matrix between climate, soil and trait principal components.** Correlation coefficient was estimated using Pearson’s *r.*

**Note S4. Random forest models specification and cross-validation**

Model specifications followed the framework provided by the ‘mlr3’ package (Lang et al., 2019), and were integrated with the package “mlr3spatiotempcv” (Schratz et al., 2023), which includes resampling methods to account for spatial autocorrelation in predictor variables. RF hyperparameters, that is the number of variables to be split at each node, minimal size of the terminal node, and the fraction of observations to be used in each tree, were tuned following a search grid approach (Probst et al., 2019) through the paradox R function (Lang et al., 2023) coupled with a spatial block resampling of the dataset. Spatial blocks are defined by bounding-boxes in the geographic space and they were defined for each individual model using the ‘blockCV’ R function (Valavi et al., 2023), which estimates the optimal box area and number of boxes to minimize the spatial autocorrelation of the predictors. We then used ten-fold spatial block partitioning, and in each of these spatial partitions we ran 100 models while using randomly selected hyperparameter configurations. The resulting model was subsequently used to predict the response variables in each hexagonal grid cell using the complete dataset per each PFT.Variable importance was assessed by estimating the average change in root mean squared error (RMSE) after permuting variables (n = 500) using the ‘DALEX’ R package (Biecek et al., 2023). This method assumes that when an important variable is permuted, this should worsen the overall model performance due to a loss of explanatory ability (i.e., RMSE loss after permutation, Breiman, 2001). Partial dependence plots were generated to visualize the marginal effects of predictors.

**References**

Biecek, P., Maksymiuk, S. & Baniecki, H. (2023). DALEX: moDel Agnostic Language for Exploration and eXplanation.

Breiman, L. (2001). Random Forests. *Machine Learning*, 45, 5–32.

Lang [cre, M., aut, Bischl, B., Richter, J., Sun, X., Binder, M., *et al.* (2023). paradox: Define and Work with Parameter Spaces for Complex Algorithms.

Lang, M., Binder, M., Richter, J., Schratz, P., Pfisterer, F., Coors, S., *et al.* (2019). mlr3: A modern object-oriented machine learning framework in R. *Journal of Open Source Software*, 4, 1903.

Probst, P., Wright, M. & Boulesteix, A.-L. (2019). Hyperparameters and Tuning Strategies for Random Forest. *WIREs Data Mining Knowl Discov*, 9.

Schratz, P., Becker, M., Muenchow, J. & Lang, M. (2023). mlr3spatiotempcv: Spatiotemporal Resampling Methods for “mlr3.”

Valavi, R., Elith, J., Lahoz-Monfort, J., Flint, I. & Guillera-Arroita, G. (2023). blockCV: Spatial and Environmental Blocking for K-Fold and LOO Cross-Validation.

**Table S5.** Effects of longitude (lon) and latitude (lat) on the residuals extracted from the random forest models (see Methods) for each STS axes and each PFTs.

| **PFT** |  | **Response** | **Est.** | **t** | ***p*** | **Adj. R^2^** |
| --- | --- | --- | --- | --- | --- | --- |
| Deciduous angiosperms | STS axis1 | Lon | 0.01 | 5.70 | <0.001 | 0.01 |
|  |  | Lat | 0.00 | -2.55 | 0.01 |  |
|  | STS axis2 | Lon | 0.00 | -1.87 | 0.06 | 0.01 |
|  |  | Lat | 0.00 | -1.15 | 0.251 |  |
|  |  |  |  |  |  |  |
| Evergreen angiosperms | STS axis1 | Lon | 0.00 | -1.62 | 0.11 | 0.00 |
|  |  | Lat | 0.00 | 0.08 | 0.94 |  |
|  | STS axis2 | Lon | 0.01 | 3.70 | <0.001 | 0.01 |
|  |  | Lat | 0.00 | -2.07 | 0.03 |  |
|  |  |  |  |  |  |  |
| Evergreen Gymnosperms | STS axis1 | Lon | 0.01 | 3.21 | 0.001 | 0.001 |
|  |  | Lat | 0.00 | -0.36 | 0.72 |  |
|  | STS axis2 | Lon | 0.00 | -0.80 | 0.42 | 0.00 |
|  |  | Lat | 0.00 | -0.91 | 0.36 |  |

Linear models results run using longitude (Lon) and latitude (Lat) as the response variables and either STS axis 1 and STS axes 2 as the independent variables. Main model statistics are shown. Est = Estimate; t = t-value, p = p-value, Adj. R^2^ = Adjusted R^2^.

**Fig. S7. Uncertainty map showing the goodness of fit of the random forest predictions for the waterlogging/cold –drought tolerance trade off.**  Log10-transformed Standard Error (SE) was used as a measure of predictions uncertainty.


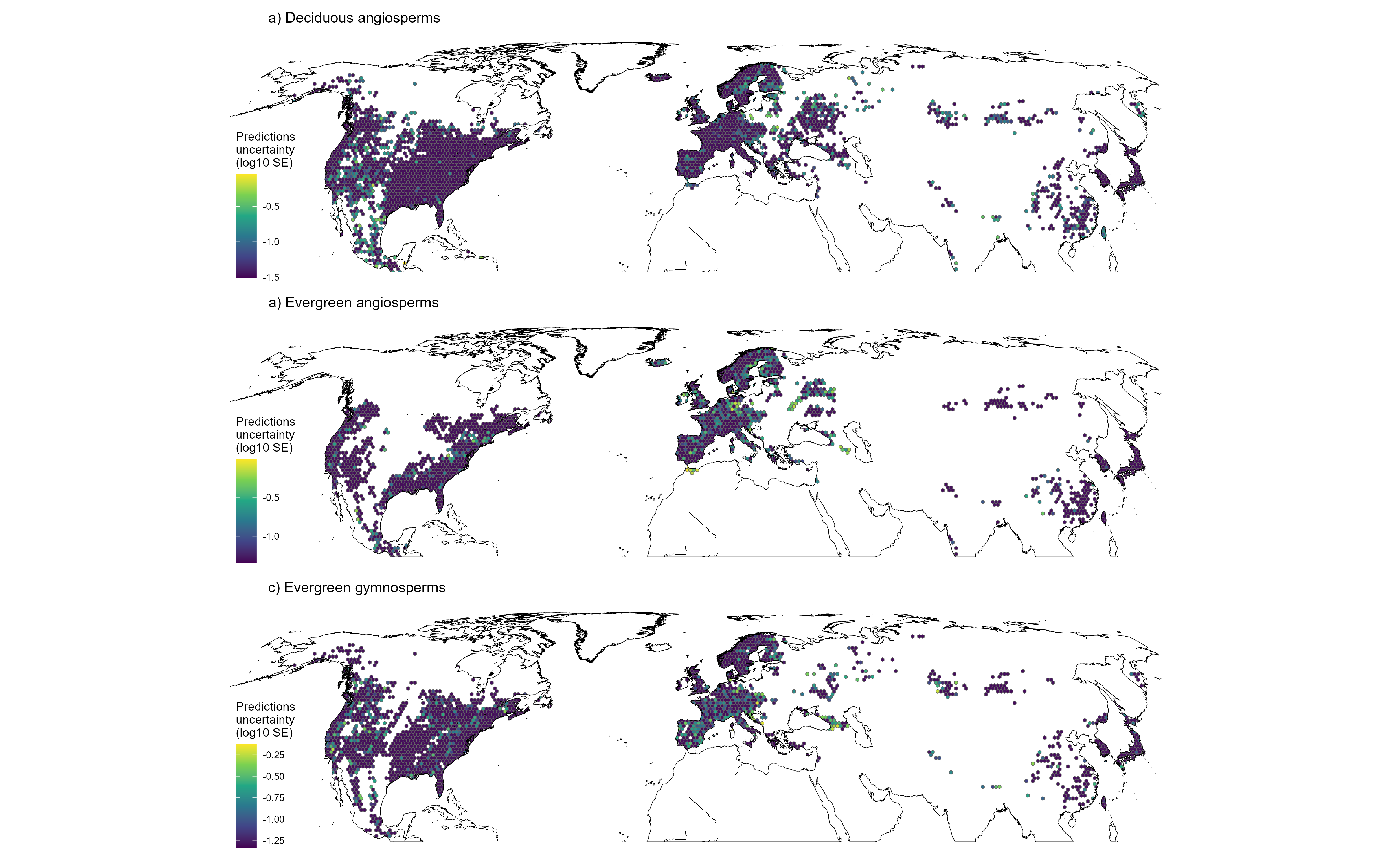


**Fig. S8. Uncertainty map showing the goodness of fit of the random forest predictions for the shade tolerance spectrum.**  Log10-transformed Standard Error (SE) was used as a measure of predictions uncertainty.


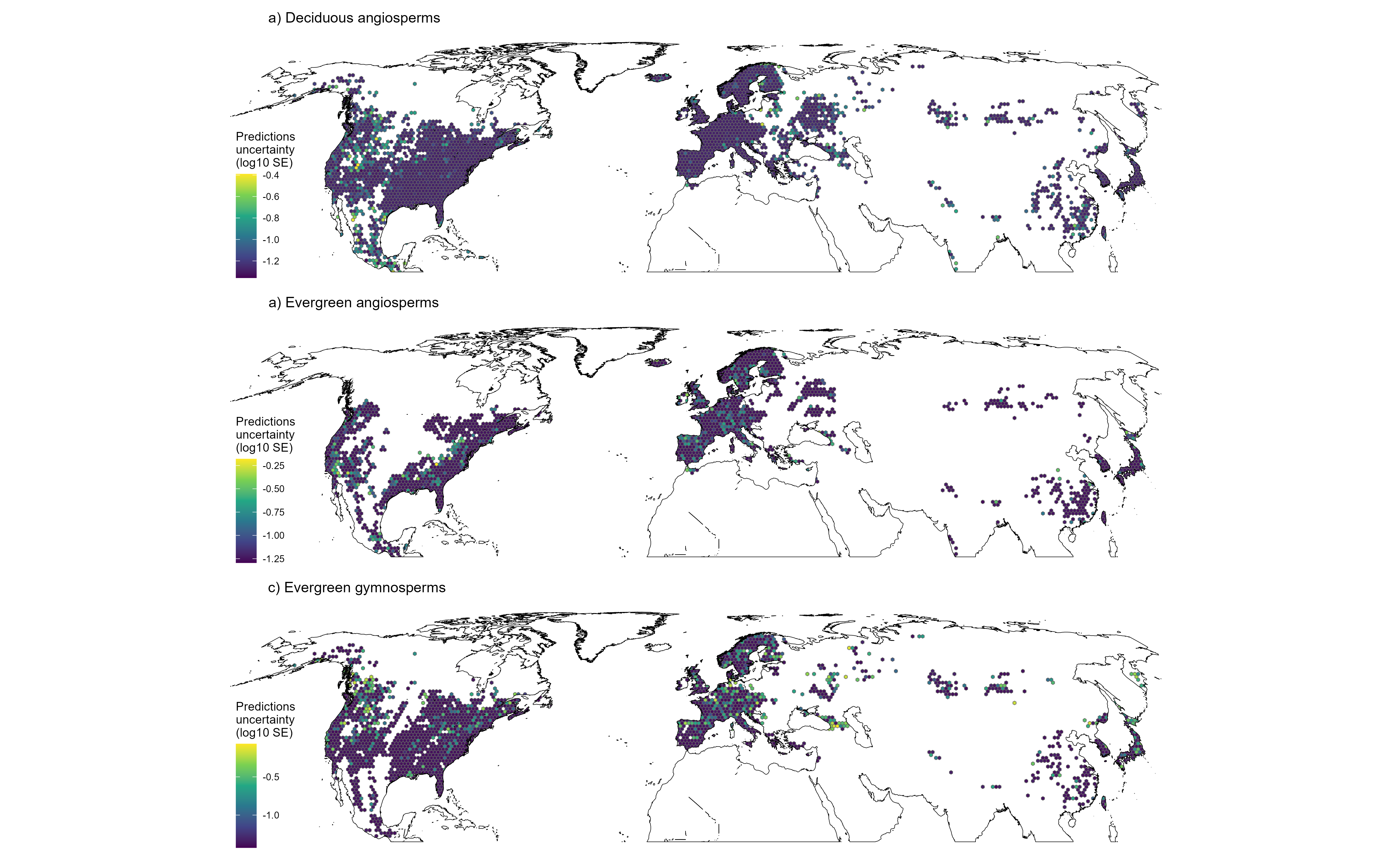

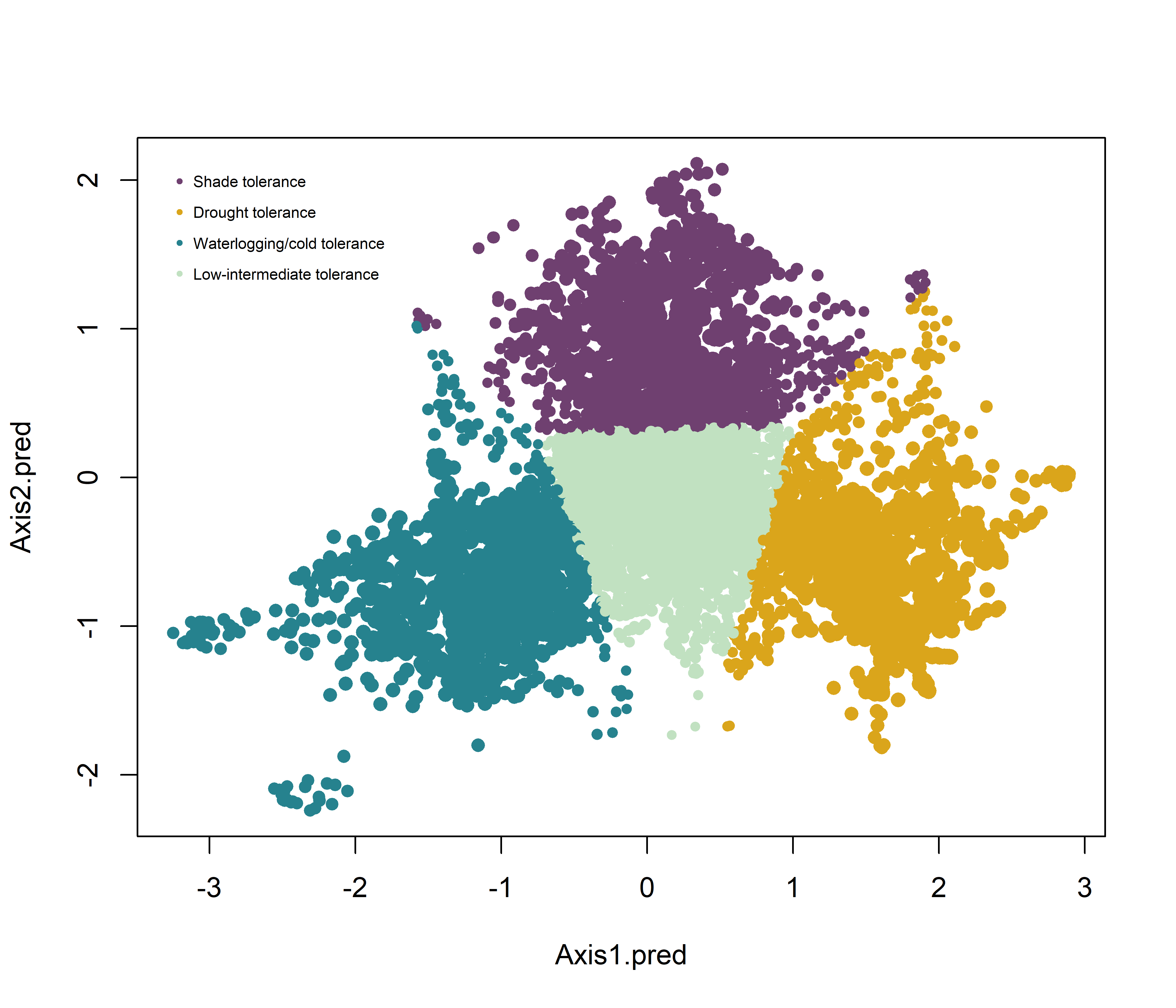


**Fig. S9. Fuzzy k-means cluster analysis using RF models predictions of the cold/wt-drought trade-off and the shade tolerance spectrum.** The four hard-clusters identified by the fuzzy k-mean cluster analysis. Each point represent a hexagon polygon of the global grid. The optimal number of clusters was estimated using the fuzzy silhouette approach (fuzzy silhouette index over 99 repetitions for k=2, 0.5858897; k=3, 0.6338040; k=4, 0.6469521; k=5, 0.6019523; k=6, 0.5028019)


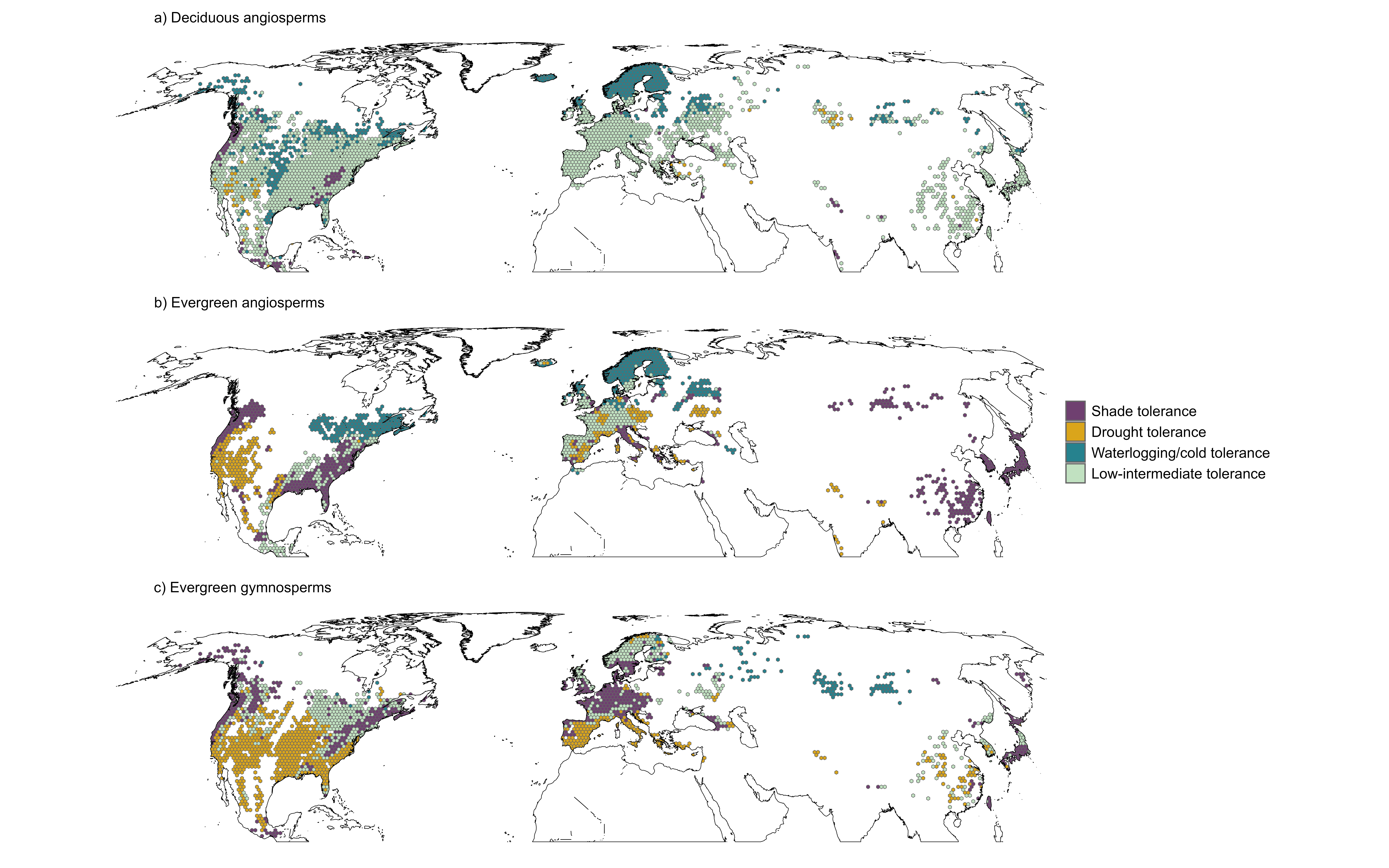


**Fig. S10. Spatial distribution of the assemblages after the after the Fuzzy k-means classification analysis** (see Fig. S9) for (a) deciduous and (b) evergreen angiosperms, and (c) evergreen gymnosperms.


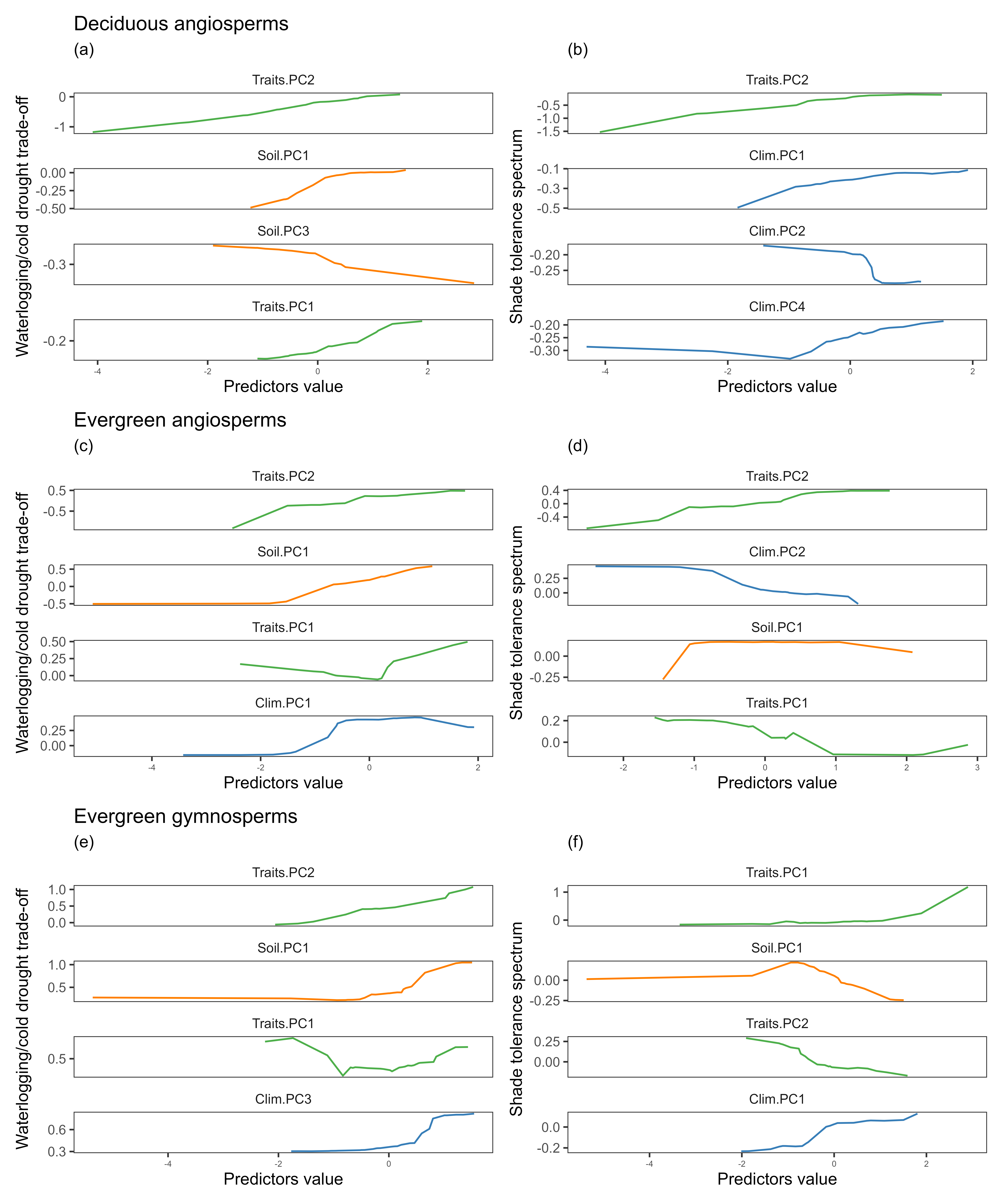


**Fig. S11. Marginal effects of the most important predictors on the Stress Tolerance Space (STS) axes**. The waterlogging/cold - drought trade-off and the shade tolerance spectrum for deciduous **(a,b)** and evergreen angiosperms **(c,d)** and evergreen gymnosperms **(e,f)**. The y axis for the waterlogging/cold – drought trade-off represents assemblages ranging from waterlogging/cold tolerant (lower values) to drought tolerant (higher values), while the y axis for the shade tolerance spectrum represents shade intolerant (lower values) to shade tolerant (higher values) assemblages. Line plots show the partial dependence plots (i.e., changes in response variable values when changing the predictor values) predicted by random forest models. Green lines indicate trait predictor, orange lines the soil predictor, and blue labels the climate predictor. All predictors have been scaled and centered before modeling.

**Table S6.** Number of assemblages (i.e. number of hexagons) associated to each of the Stress Tolerance Biomes (STB) and polytolerance hotspots, for each plant functional type. In parenthesis, the relative value to the total number of hexagons for that plant functional type.

| **Stress tolerance Biome** | **Deciduous Angiosperms** | **Evergreen Angiosperms** | **Evergreen Gymnosperms** |
| --- | --- | --- | --- |
| Drought STB | 29 (1%) | 220 (11.4%) | 746 (33.2%) |
| Shade STB | 179 (6.4%) | 510 (26.5%) | 587 (26.1%) |
| Waterlogging/cold STB | 547 (19.5%) | 408 (21.2%) | 65 (2.9%) |
| **Polytolerance Hotspots** |  |  |  |
| Shade-drought polytolerance hotspot | 38 (1.4%) | 74 (3.8%) | 26 (1.1%) |
| Shade-waterlogging/cold polytolerance hotspot | 142 (5%) | 17 (0.8%) | 30 (1.3%) |
|  |  |  |  |
| **Low-intermediate tolerance assemblages** | 1856 (66.4%) | 690 (36%) | 791 (35.2%) |
|  |  |  |  |

**
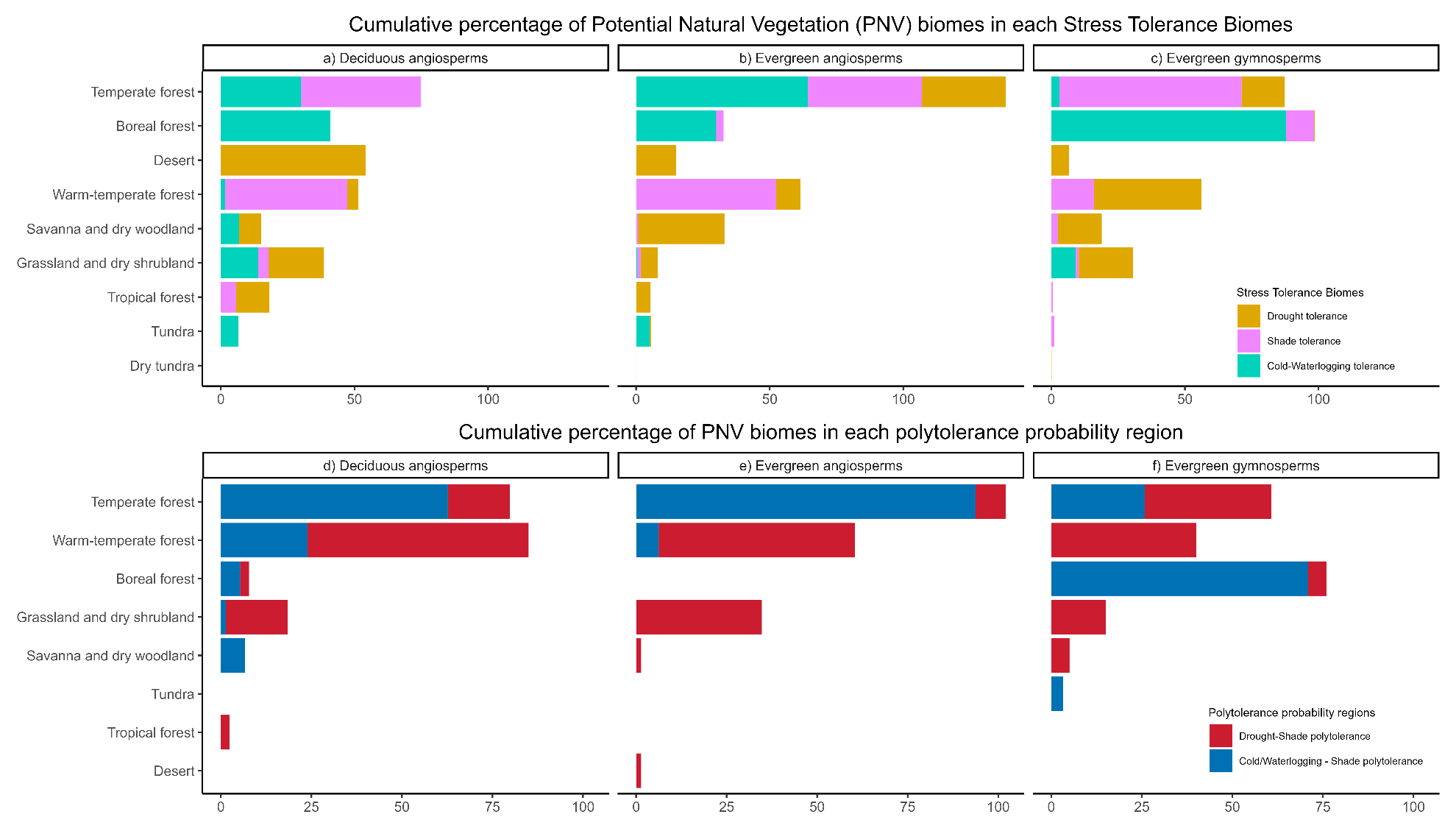
**

**Fig. S12. Main Potential Natural Vegetation (PNV) biomes associated to each Stress Tolerance Biome (STB) and polytolerance hotspots**, for deciduous and evergreen angiosperms (a-b, d-e) and evergreen gymnosperms (c, f). The percentage of each PNV biome associated with the STBs and polytolerance hotspot was estimated by linking each assemblage with the predominant PNV biome covering that assemblage.


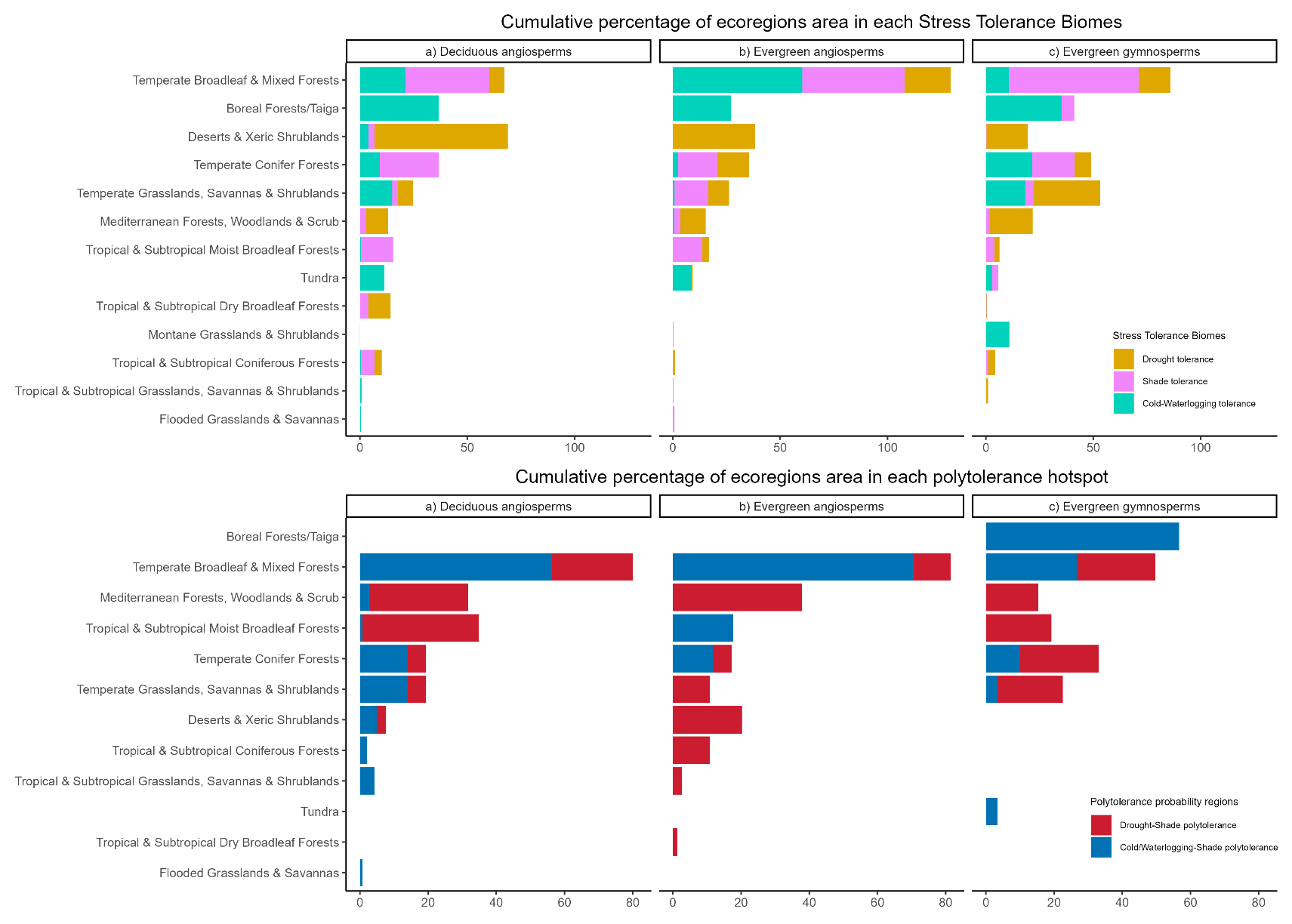


**Fig. S13. Ecoregions biomes associated to each Stress Tolerance Biome (STB) and polytolerance hotspots**, for deciduous and evergreen angiosperms (a-b, d-e) and evergreen gymnosperms (c, f). The percentage of each PNV biome associated with the STBs and polytolerance hotspot was estimated by linking each assemblage with the predominant ecoregion biome covering that assemblage. Ecoregions classification follows Dinerstein et al. (2017).

**
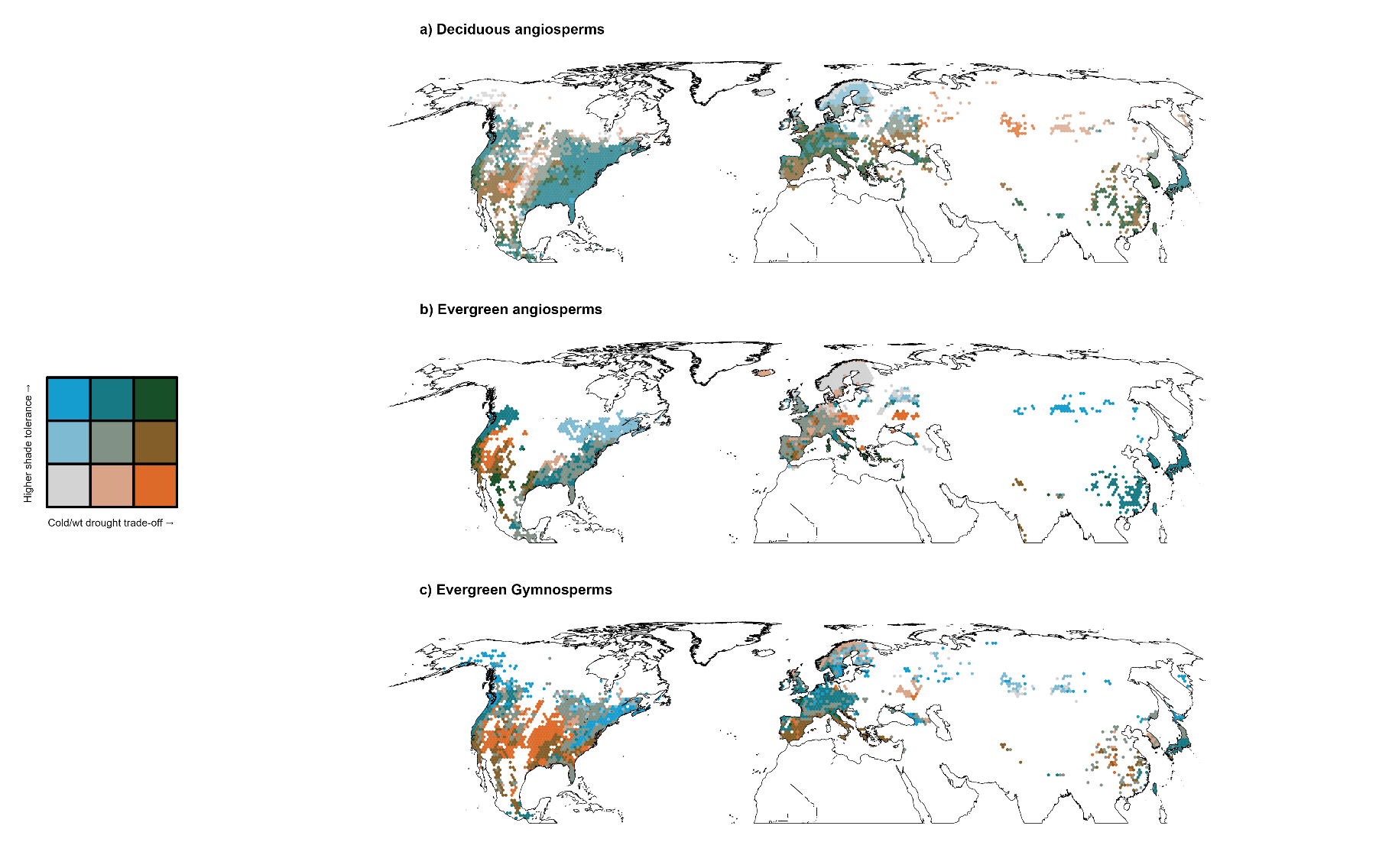
**

**Fig. S14. Abiotic stress tolerance strategies in the geographic space.** Bivariate choropleth maps showing the combinations of the waterlogging/cold - drought tolerance trade-off and the shade tolerance spectrum values for **(a)** deciduous angiosperms, **(b)** evergreen angiosperms and **(c)** evergreen gymnosperms. Each hexagon has an area of 7500 km^2^. Orange hexagons correspond to the drought tolerance strategy. Azure-dark green hexagons indicate the dominance of shade tolerance strategies. Grey hexagons correspond to cold-waterlogging tolerance strategies. Intermediate colors indicate areas of relative polytolerance between different stresses. The classes corresponding to each color were calculated separately for each PFT to highlight intra-group specific differences in stress tolerance strategies





**Fig. S15. The drought Stress Tolerance Biome (STB) inside the climate, soil and traits spaces,** for deciduous (a) and evergreen (b) angiosperms and evergreen gymnosperms (c).

Kernel density estimation (i.e., probability) for the drought STB mapped inside the space defined by the first two dimension of the climate, soil and functional traits PCAs (see Fig. S3, S4, S5). Grey points represent the hexagons belonging to the drought STB after the classification detailed in main text (see Methods - Defining the Stress Tolerance Biomes and polytolerance hotspots). Contour lines indicate the 0.99, 0.75 and 0.50 quantile of the total probability distribution. The colors represent the probability distribution, ranging from high (dark yellow) to low (light yellow).





**Fig. S16. The shade Stress Tolerance Biome (STB) inside the climate, soil and traits spaces,** for deciduous (a) and evergreen (b) angiosperms and evergreen gymnosperms (c).

Kernel density estimation (i.e., probability) for the shade STB mapped inside the space defined by the first two dimension of the climate, soil and functional traits PCAs (see Fig. S3, S4, S5). Grey points represent the hexagons belonging to the shade STB after the classification detailed in main text (see Methods - Defining the Stress Tolerance Biomes and polytolerance hotspots). Contour lines indicate the 0.99, 0.75 and 0.50 quantile of the total probability distribution. The colors represent the probability distribution, ranging from high (dark magenta) to low (light magenta).





**Fig. S17. The waterlogging/cold Stress Tolerance Biome (STB) inside the climate, soil and traits spaces,** for deciduous (a) and evergreen (b) angiosperms and evergreen gymnosperms (c).

Kernel density estimation (i.e., probability) for the waterlogging/cold STB mapped inside the space defined by the first two dimension of the climate, soil and functional traits PCAs (see Fig. S3, S4, S5). Grey points represent the hexagons belonging to the waterlogging/cold STB after the classification detailed in main text (see Methods - Defining the Stress Tolerance Biomes and polytolerance hotspots). Contour lines indicate the 0.99, 0.75 and 0.50 quantile of the total probability distribution. The colors represent the probability distribution, ranging from high (dark cyan) to low (light cyan).





**Fig. S18. The shade-drought polytolerance hotspot inside the climate, soil and traits spaces,** for deciduous (a) and evergreen (b) angiosperms and evergreen gymnosperms (c).

Kernel density estimation (i.e., probability) for the shade-drought polytolerance hotspot mapped inside the space defined by the first two dimension of the climate, soil and functional traits PCAs (see Fig. S3, S4, S5). Grey points represent the hexagons belonging to the shade-drought polytolerance hotspot after the classification detailed in main text (see Methods - Defining the Stress Tolerance Biomes and polytolerance hotspots). Contour lines indicate the 0.99, 0.75 and 0.50 quantile of the total probability distribution. The colors represent the probability distribution, ranging from high (dark red) to low (light red).





**Fig. S19. The shade- waterlogging/cold polytolerance hotspot inside the climate, soil and traits spaces,** for deciduous (a) and evergreen (b) angiosperms and evergreen gymnosperms (c).

Kernel density estimation (i.e., probability) for the waterlogging/cold polytolerance hotspot mapped inside the space defined by the first two dimension of the climate, soil and functional traits PCAs (see Fig. S3, S4, S5). Grey points represent the hexagons belonging to the waterlogging/cold polytolerance hotspot after the classification detailed in main text (see Methods - Defining the Stress Tolerance Biomes and polytolerance hotspots). Contour lines indicate the 0.99, 0.75 and 0.50 quantile of the total probability distribution. The colors represent the probability distribution, ranging from high (dark blue) to low (light blue)

**Table S7.** Most abundant species in each Potential Natural Vegetation (PNV) biome associated with each Stress Tolerance Biome (STB) and polytolerance hotspot.

| **PNV Biome** | **Deciduous angiosperms** | **Evergreen angiosperms** | **Evergreen gymnosperms** |
| --- | --- | --- | --- |
| **Drought STB** | | | |
| Desert | *Chilopsis linearis, Quercus gambelii, Robinia neomexicana, Populus fremontii, Fraxinus velutina, Quercus × undulata, Juglans major, Populus tremuloides, Quercus muehlenbergii, Ptelea trifoliata* | *Yucca brevifolia, Quercus turbinella, Cercocarpus ledifolius, Quercus chrysolepis, Prunus ilicifolia, Purshia mexicana, Quercus emoryi, Quercus wislizeni, Umbellularia californica, Quercus dumosa* | *Pinus monophylla, Juniperus deppeana, Pinus edulis, Pinus ponderosa, Juniperus monosperma, Juniperus osteosperma, Cupressus arizonica, Abies concolor, Pseudotsuga menziesii, Pinus strobiformis* |
| Grassland and dry shrubland | *Chilopsis linearis, Juglans major, Populus fremontii, Prosopis juliflora, Fraxinus velutina, Prunus serotina, Celtis laevigata* | *Prunus ilicifolia, Quercus chrysolepis, Yucca brevifolia, Quercus wislizeni, Quercus agrifolia, Cercocarpus ledifolius, Helianthemum apenninum, Heteromeles arbutifolia, Umbellularia californica, Quercus dumosa* | *Pinus coulteri, Juniperus deppeana, Calocedrus decurrens, Pseudotsuga macrocarpa, Pinus monophylla, Juniperus virginiana, Pinus halepensis, Abies concolor, Pinus jeffreyi, Cupressus arizonica* |
| Savanna and dry woodland | *Elaeagnus angustifolia, Tamarix ramosissima, Albizia julibrissin, Cotinus coggygria, Prunus armeniaca, Ziziphus jujuba, Prunus persica, Ailanthus altissima, Morus alba, Pistacia vera* | *Cercocarpus ledifolius, Quercus turbinella, Berberis aquifolium, Yucca brevifolia, Purshia mexicana, Quercus chrysolepis, Umbellularia californica, Platanus × hispanica, Quercus emoryi* | *Pinus edulis, Juniperus osteosperma, Pinus flexilis, Juniperus scopulorum, Pinus ponderosa, Pinus monophylla, Juniperus monosperma, Picea pungens, Pseudotsuga menziesii, Abies concolor* |
| Temperate forest |  | *Hyssopus officinalis ,Genista pilosa, ,Helianthemum apenninum ,Fumana procumbens ,Helianthemum nummularium, ,Thymus serpyllum, Daphne laureola, Buxus sempervirens,Daphne cneorum ,Quercus ilex* | *Pinus nigra ,Juniperus virginiana ,Pinus halepensis,Pinus uncinata, Juniperus communis var. saxatilis,Cupressus Juniperus communis,Pinus echinata, ,Juniperus,Pinus densiflora* |
| Tropical forest | *Prosopis juliflora, Liquidambar styraciflua* | *Hibiscus rosa-sinensis* |  |
| Warm-temperate forest | *Melia azedarach, Ailanthus altissima, Albizia julibrissin, Pistacia vera, Cydonia oblonga, Prunus armeniaca, Elaeagnus angustifolia, Morus alba, Platanus orientalis, Ziziphus jujuba* | *Helianthemum apenninum, Fumana procumbens, Hyssopus officinalis, Arctostaphylos uva-ursi, Arbutus unedo, Quercus ilex, Buxus sempervirens, Cytisus scoparius, Ilex aquifolium, Eriobotrya japonica* | *Pinus halepensis, Pinus palustris, Cupressus sempervirens, Pinus taeda, Juniperus virginiana, Pinus nigra, Pinus echinata, Pinus elliottii, Juniperus deppeana, Juniperus virginiana var. silicicola* |
| Tundra |  | *Thymus serpyllum* |  |
| Dry tundra |  |  | *Cupressus sempervirens, Pinus halepensis* |
| Boreal forest |  |  | *Pinus albicaulis, Juniperus sabina, Juniperus scopulorum, Pinus monticola, Abies lasiocarpa, Pinus contorta, Pinus contorta var. latifolia, Pseudotsuga menziesii* |
| **Shade STB** | | | |
| Grassland and dry shrubland | *Acer macrophyllum, Rhododendron occidentale, Melia azedarach, Aesculus californica, Sambucus cerulea, Corylus cornuta, Ailanthus altissima, Rosa californica, Cornus sericea, Acer negundo* | *Eriobotrya japonica, Rhododendron ponticum, Arbutus unedo, Calluna vulgaris, Ulex minor, Erica erigena,europaeus, Erica ciliaris, Berberis aquifolium, Ilex aquifolium* | *Sequoia sempervirens, Pinus radiata, Torreya californica, Pseudotsuga menziesii, Pinus attenuata, Calocedrus decurrens, Pinus sabiniana, Pinus muricata, Pinus coulteri, Abies grandis* |
| Savanna and dry woodland |  | *Sabal palmetto, Berberis aquifolium, Sideroxylon reclinatum, Ilex cassine, Morella cerifera, Quercus virginiana, Sideroxylon celastrinum, Persea borbonia* | *Picea engelmannii, Pinus ponderosa, Pseudotsuga menziesii, Abies grandis, Abies lasiocarpa, Thuja plicata, Taxus brevifolia, Picea pungens, Juniperus scopulorum, Pinus albicaulis* |
| Temperate forest | *Acer macrophyllum, Rubus spectabilis, Alnus rubra, Magnolia fraseri, Acer circinatum, Acer pensylvanicum, Frangula purshiana, Oxydendrum arboreum, Euonymus americanus, Sassafras albidum* | *Rhododendron maximum, Gaultheria shallon, Kalmia latifolia, Leucothoe grayana, Berberis aquifolium, Arbutus menziesii, Ilex opaca, Aucuba japonica, Skimmia japonica, Prunus laurocerasus* | *Abies alba, Thuja plicata, Tsuga canadensis, Taxus baccata, Picea abies, Pseudotsuga menziesii, Picea rubens, Pinus mugo, Pinus nigra, Pinus strobus* |
| Tropical forest | *Ostrya virginiana, Tilia americana, Lagerstroemia indica, Prosopis juliflora, Liquidambar styraciflua, Prunus serotina, Melia azedarach, Carpinus caroliniana, Juglans major, Salix discolor* |  | *Pinus strobus* |
| Warm-temperate forest | *Quercus garryana, Rhododendron occidentale, Acer macrophyllum, Ostrya virginiana, Liquidambar styraciflua, Prunus serotina, Carpinus caroliniana, Cornus nuttallii, Corylus cornuta, Acer circinatum* | *Sabal palmetto, Quercus glauca, Vaccinium arboreum, Ilex opaca, Machilus thunbergii, Ilex vomitoria, Prunus caroliniana, Quercus virginiana, Machilus japonica, Magnolia grandiflora* | *Sequoia sempervirens, Pseudotsuga menziesii, Calocedrus decurrens, Cryptomeria japonica, Chamaecyparis obtusa, Taxus brevifolia, Chamaecyparis lawsoniana, Pinus ponderosa, Pinus lambertiana, Pinus jeffreyi* |
| Boreal Forest |  | *Berberis aquifolium, Chimaphila umbellata* | *Taxus baccata, Abies lasiocarpa, Picea glauca, Abies alba, Picea abies, Pinus sylvestris, Thuja plicata, Pinus contorta, Pseudotsuga menziesii, Juniperus communis* |
| Dry tundra |  | *Ligustrum lucidum* |  |
| Tundra |  |  | *Tsuga mertensiana, Picea glauca, Picea sitchensis, Abies lasiocarpa, Picea mariana, Pinus contorta, Pinus contorta var. latifolia* |
| **Waterlogging/Cold STB** | | | |
| Boreal forest | *Salix lapponum, Betula nana, Salix phylicifolia, Salix myrsinifolia, Salix myrsinites, Salix herbacea, Salix aurita, Salix myrtilloides, Vaccinium myrtillus, Ribes spicatum* | *Empetrum nigrum subsp. hermaphroditum, Vaccinium microcarpum, Linnaea borealis, Vaccinium vitis-idaea, Vaccinium uliginosum, Empetrum nigrum, Myrica gale, Erica tetralix, Calluna vulgaris, Arctostaphylos uva-ursi* | *Pinus sibirica, Abies sibirica, Picea obovata, Pinus sylvestris* |
| Grassland and dry shrubland | *Populus deltoides, Populus deltoides subsp. monilifera, Salix amygdaloides, Fraxinus pennsylvanica, Rosa arkansana, Salix nigra, Celtis laevigata, Celtis occidentalis, Gleditsia triacanthos, Prunus virginiana* | *Ledum palustre, Prunus lusitanica* | *Pinus sibirica, Picea obovata* |
| Savanna and dry woodland | *Populus tremuloides, Prunus virginiana, Populus angustifolia, Salix bebbiana, Salix amygdaloides, Betula occidentalis, Populus deltoides, Populus deltoides subsp. monilifera, Shepherdia argentea, Populus × acuminata* |  |  |
| Temperate forest | *Salix herbacea, Salix aurita, Salix repens, Salix phylicifolia, Rosa sherardii, Vaccinium myrtillus, Acer pseudoplatanus, Sorbus aucuparia, Salix cinerea, Ulmus glabra* | *Myrica gale, Erica cinerea, Kalmia angustifolia, Erica tetralix, Ulex europaeus, Empetrum nigrum, Ilex aquifolium, Vaccinium vitis-idaea, Empetrum nigrum subsp. hermaphroditum, Rhododendron ponticum* | *Pinus sibirica* |
| Tundra | *Salix lapponum, Salix herbacea, Betula pubescens, Salix phylicifolia, Betula nana, Salix hastata, Alnus incana, Salix lanata, Salix caprea, Sorbus aucuparia* | *Empetrum nigrum subsp. hermaphroditum, Vaccinium vitis-idaea, Empetrum nigrum, Linnaea borealis, Vaccinium uliginosum, Calluna vulgaris, Vaccinium microcarpum, Veronica fruticans, Erica tetralix, Dryas octopetala* |  |
| Warm-temperate forest | *Cephalanthus occidentalis, Celtis laevigata, Salix nigra, Populus deltoides, Liquidambar styraciflua, Zanthoxylum clava-herculis, Gleditsia triacanthos, Ulmus crassifolia, Clethra alnifolia, Carya illinoinensis* |  |  |
| ***Shade – Drought Politolerance Hotspot*** | | | |
| Boreal forest | *Cytisus hirsutus, Rhamnus alpina, Rhamnus pumila, Salix alba, Sorbus arranensis, Acer monspessulanum* |  |  |
| Grassland and dry shrubland | *Quercus douglasii, Quercus lobata, Quercus kelloggii, Rosa californica, Acer macrophyllum, Aesculus californica, Platanus orientalis, Populus fremontii, Sambucus cerulea, Symphoricarpos albus* | *Quercus emoryi, Notholithocarpus densiflorus, Arbutus menziesii, Arbutus unedo, Eriobotrya japonica, Umbellularia californica, Heteromeles arbutifolia, Quercus agrifolia, Helianthemum apenninum, Chrysolepis chrysophylla* | *Sequoiadendron giganteum, Pinus sabiniana, Calocedrus decurrens, Pinus lambertiana, Abies magnifica, Abies concolor, Sequoia sempervirens, Torreya californica, Pinus jeffreyi, Pinus ponderosa* |
| Temperate forest | *Euonymus verrucosus, Cotinus coggygria, Cornus mas, Euonymus latifolius, Elaeagnus angustifolia, Lycium barbarum, Tamarix ramosissima, Berberis vulgaris, Prunus armeniaca, Fagus orientalis* | *Daphne laureola, Rhododendron ferrugineum, Helianthemum nummularium, Ilex aquifolium, Prunus laurocerasus, Buxus sempervirens, Fumana procumbens, Vaccinium vitis-idaea, Arctostaphylos uva-ursi, Arbutus unedo* | *Pinus cembra, Juniperus communis var. saxatilis, Pinus mugo, Abies alba, Pinus uncinata, Juniperus communis, Picea abies, Juniperus sabina, Cupressus sempervirens, Taxus baccata* |
| Tropical forest | *Lagerstroemia indica, Albizia julibrissin, Melia azedarach, Prunus persica* |  |  |
| Warm-temperate forest | *Platanus orientalis, Cotinus coggygria, Prunus armeniaca, Juglans regia, Cydonia oblonga, Colutea arborescens, Quercus pubescens, Hippocrepis emerus, Prunus cerasifera, Symplocos paniculata* | *Heteromeles arbutifolia, Arbutus menziesii, Umbellularia californica, Ilex vomitoria, Arbutus unedo, Quercus ilex, Quercus agrifolia, Eriobotrya japonica, Quercus wislizeni, Helianthemum apenninum* | *Pinus glabra, Pinus echinata, Pinus palustris, Pinus taeda, Pinus sylvestris, Cupressus sempervirens, Juniperus virginiana, Juniperus sabina, Taxus baccata, Juniperus communis var. saxatilis* |
| Desert |  | *Quercus emoryi* |  |
| Savanna and dry woodland |  |  | *Pinus jeffreyi, Calocedrus decurrens, Juniperus occidentalis, Abies magnifica, Abies concolor, Pinus lambertiana, Pinus monticola, Tsuga mertensiana, Pinus contorta, Pinus albicaulis* |
| **Shade – Waterlogging/cold Polytolerance Hotspot** | | | |
| Boreal forest | *Amelanchier alnifolia, Acer glabrum, Prunus cerasus, Symphoricarpos albus, Alnus alnobetula subsp. sinuata, Quercus petraea, Betula papyrifera, Cornus sericea, Tilia × europaea, Acer pseudoplatanus* |  | *Abies sibirica, Picea mariana, Picea obovata, Picea glauca, Picea abies, Pinus sylvestris, Juniperus communis, Pinus banksiana, Juniperus communis var. saxatilis* |
| Grassland and dry shrubland | *Ulmus crassifolia, Celtis laevigata, Platanus occidentalis, Zanthoxylum clava-herculis, Salix nigra, Ptelea trifoliata, Ilex decidua, Aesculus pavia, Cephalanthus occidentalis, Chilopsis linearis* |  |  |
| Savanna and dry woodland | *Amelanchier alnifolia, Acer glabrum, Prunus virginiana, Symphoricarpos albus, Betula occidentalis, Sambucus cerulea, Cornus sericea, Sorbus scopulina, Swietenia mahagoni, Symphoricarpos oreophilus* |  |  |
| Temperate forest | *Betula alleghaniensis, Populus grandidentata, Acer pensylvanicum, Betula populifolia, Betula lenta, Ilex verticillata, Acer spicatum, Hamamelis virginiana, Acer saccharum, Rhus typhina* | *Kalmia angustifolia, Prunus laurocerasus, Ledum palustre, Ulex gallii, Kalmia latifolia, Ilex opaca, Ulex europaeus, Rhododendron ponticum, Myrica gale, Vaccinium macrocarpon* | *Pinus contorta, Picea mariana, Picea sitchensis, Pinus banksiana, Abies sibirica, Thuja occidentalis, Cupressus nootkatensis, Pinus resinosa, Tsuga mertensiana* |
| Warm-temperate forest | *Quercus nigra, Magnolia virginiana, Rhus copallinum, Celtis laevigata, Diospyros virginiana, Aesculus pavia, Cephalanthus occidentalis, Cyrilla racemiflora, Platanus occidentalis, Liquidambar styraciflua* | *Morella cerifera* |  |
| Tundra |  |  | *Picea obovata* |

**
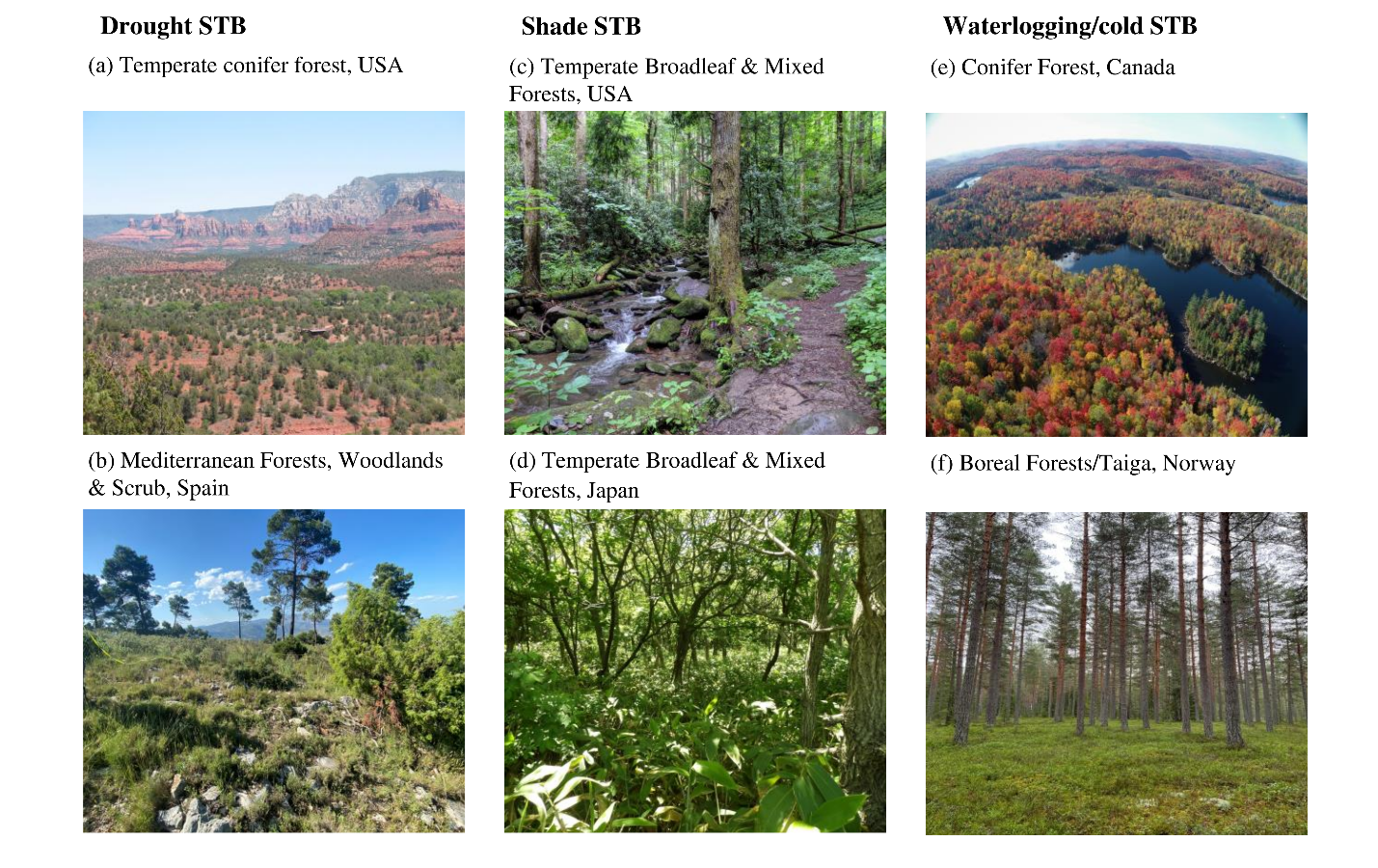

Fig. S20. Representative cases of natural vegetation for each stress tolerance biomes (STB)**. Drought STB: (a) Pinyon-juniper-cypress woodland in the Red Rock State Park, Sedona (Arizona, United States), (b) Garrigue in Parque Natural del Carrascal de la Font Roja (Comunidad Valenciana, Spain). Shade STB: (c) Temperate broadleaf blue ridge forest in the Great Smokey Mountains National Park (Tennessee, United States), (d) Coastal oak woodland in the Ishikari plain (Hokkaido, Japan); Waterlogging/cold STB: (e) Subhumid Eastern Temperate Hardwood from the Station de biologie des Laurentides (SBL) de l'Université de Montréal (Québec, Canada), (f) Temperate continental Pinus sylvestris forest in Vikersund (Buskerud, Norway).

Open access photos are available from the Global Vegetation Project (<http://gveg.wyobiodiversity.org> ) (Fleri et al., 2021) and reproduced under the CC-BY-NC-SA 4.0 license.

**
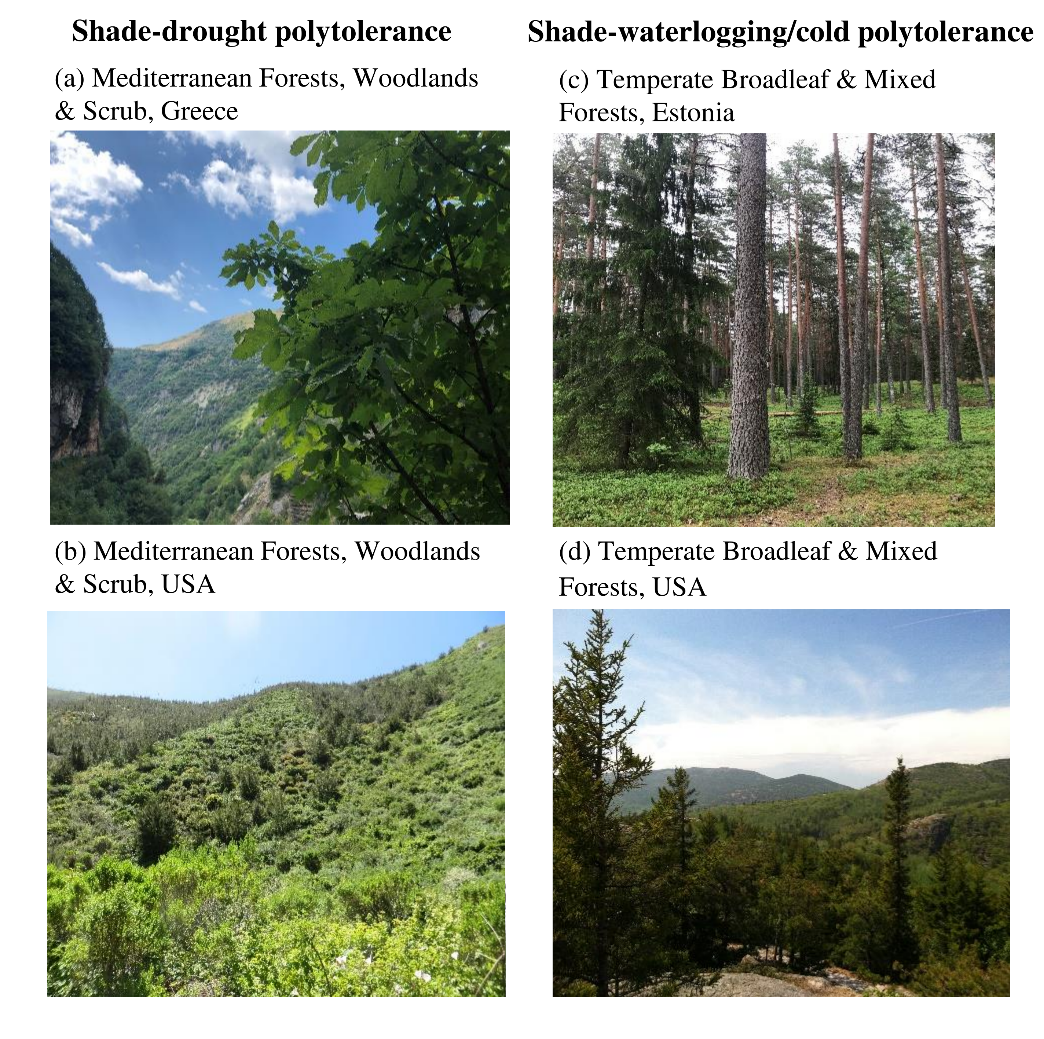
**

**Fig. S21. Representative cases of natural vegetation for each polytolerance hotspot.** Shade-Drought polytolerance hotspot: **(a)** Thermophilous deciduous woodland near Syrrako (Epirus and Western Macedonia, Greece), **(b)** Coastal scrub in Coon Creek Trail, Montaña de Oro State Park (California, United States). Shade-waterlogging/cold polytolerance hotspot: **(c)** Hemiboreal pine forest in the Meenikunno hiking trail (Põlva, Estonia), **(d)** Laurentian-Acadian Hemlock - White Pine - Hardwood Forest in the Acadia National Park (Maine, United States)

Open access photos are available from the Global Vegetation Project (<http://gveg.wyobiodiversity.org>) (Fleri et al., 2021) and reproduced under the CC-BY-NC-SA 4.0 license.
